# Emerging biomaterials for tumor immunotherapy

**DOI:** 10.1186/s40824-023-00369-8

**Published:** 2023-05-16

**Authors:** Minna Xiao, Qinglai Tang, Shiying Zeng, Qian Yang, Xinming Yang, Xinying Tong, Gangcai Zhu, Lanjie Lei, Shisheng Li

**Affiliations:** 1 Department of Otorhinolaryngology Head and Neck Surgery, The Second Xiangya Hospital, Central South University, Changsha, 410011 Hunan China; 2 Department of Hemodialysis, the Second Xiangya Hospital, Central South University, Changsha, 410011 Hunan China; 3 State Key Laboratory of Bioelectronics, School of Biological Science and Medical Engineering, Southeast University, Nanjing, 210096 China

**Keywords:** Tumor immunotherapy, Biomaterial, Nanoparticles, Liposomes, Cell membranes

## Abstract

**Background:**

The immune system interacts with cancer cells in various intricate ways that can protect the individual from overproliferation of cancer cells; however, these interactions can also lead to malignancy. There has been a dramatic increase in the application of cancer immunotherapy in the last decade. However, low immunogenicity, poor specificity, weak presentation efficiency, and off-target side effects still limit its widespread application. Fortunately, advanced biomaterials effectively contribute immunotherapy and play an important role in cancer treatment, making it a research hotspot in the biomedical field.

**Main body:**

This review discusses immunotherapies and the development of related biomaterials for application in the field. The review first summarizes the various types of tumor immunotherapy applicable in clinical practice as well as their underlying mechanisms. Further, it focuses on the types of biomaterials applied in immunotherapy and related research on metal nanomaterials, silicon nanoparticles, carbon nanotubes, polymer nanoparticles, and cell membrane nanocarriers. Moreover, we introduce the preparation and processing technologies of these biomaterials (liposomes, microspheres, microneedles, and hydrogels) and summarize their mechanisms when applied to tumor immunotherapy. Finally, we discuss future advancements and shortcomings related to the application of biomaterials in tumor immunotherapy.

**Conclusion:**

Research on biomaterial-based tumor immunotherapy is booming; however, several challenges remain to be overcome to transition from experimental research to clinical application. Biomaterials have been optimized continuously and nanotechnology has achieved continuous progression, ensuring the development of more efficient biomaterials, thereby providing a platform and opportunity for breakthroughs in tumor immunotherapy.

**Graphical Abstract:**

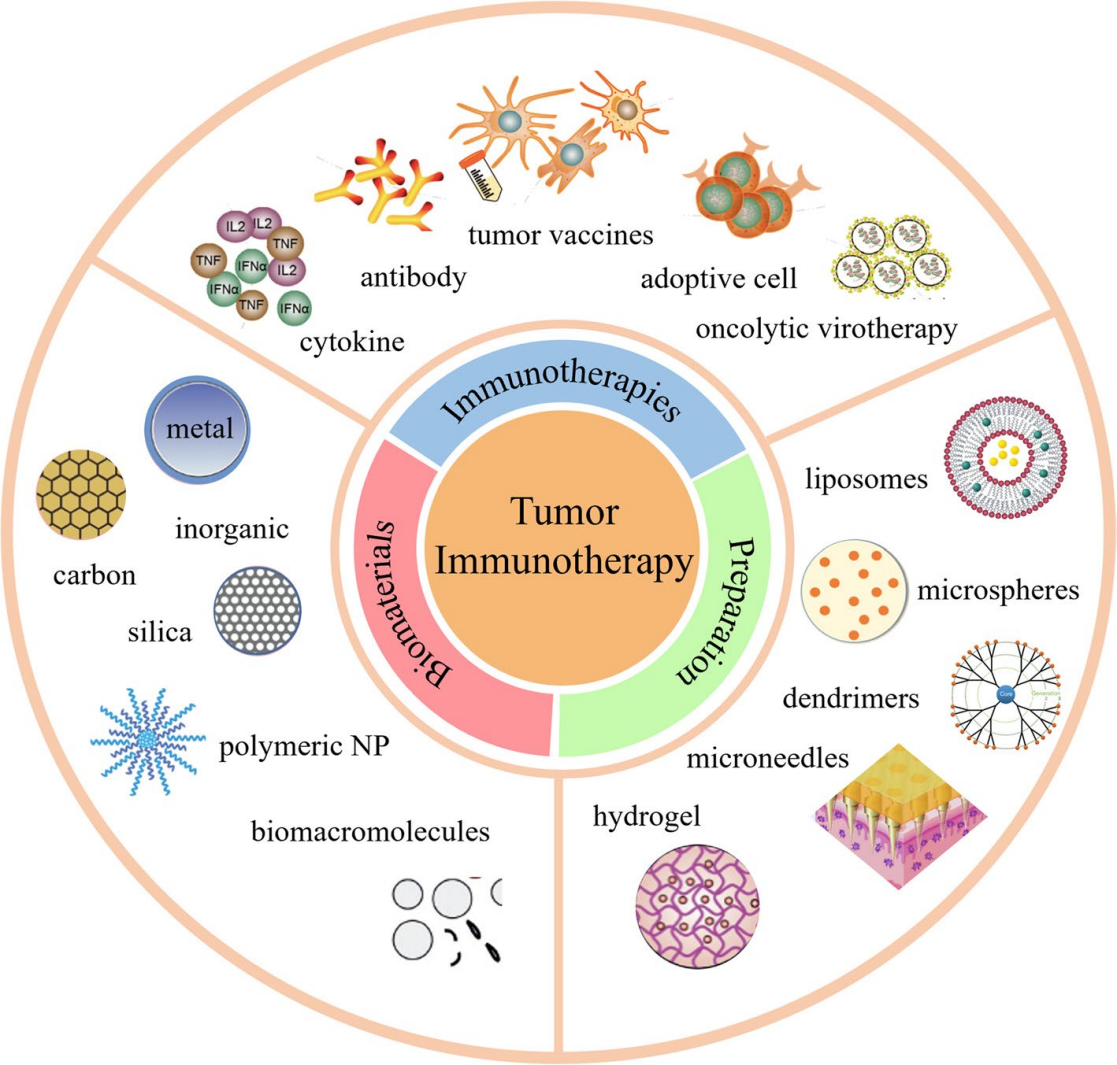

## Background

The increase in the size and age of the adult population is accompanied by increased cancer incidence and mortality; in addition, tumors have become a major cause of death globally [1]. Researchers have focused on developing effective treatments for tumors. Recent treatment approaches for tumors include surgery, chemotherapy, immunotherapy, radiotherapy, and targeted therapy. Various treatment methods have made some progress in the elimination of tumor cells [1]. Surgery is the major treatment for cancer; however, it can only remove solid tumors. For scattered tumor cells, chemotherapy and radiotherapy are the appropriate treatment options. Immunotherapy for cancer treatment acts by killing tumor cells that depend on the body’s immune system [2]. Recently, there have rapid developments of antibody drugs and biological technologies; furthermore, disciplines of oncology, immunology, molecular biology, and others have achieved cross-penetration; moreover, the tumor immune escape mechanism and tumor microenvironment (TME) have been widely explored. All these advancements have improved tumor immunotherapy strategies. Immunotherapy provides a new therapeutic approach besides surgery, conventional chemotherapy, and radiotherapy. Its practical application embodies advantages that cannot be compared with those of traditional therapies, such as reduced damage to organs, fewer side effects, less likelihood of resultant drug resistance, stronger effectiveness in removing residual tumor cells, long-term effectiveness, and prevention of recurrence [3]. However, immunotherapy still has certain limitations. Poor specificity, low immunogenicity, insufficient delivery efficiency, and off-target side effects its main challenges. To address these, researchers have recently begun working on the application of biomaterials in tumor immunotherapy, as they demonstrate superiority in the delivery and precise modulation of immunotherapy. Compared to conventional immunotherapeutic agents, biomaterials can prolong circulation time, improve pharmacokinetic parameters, target delivery, promote uptake and presentation, and control release [4]. This review discusses various types of tumor immunotherapy and their underlying mechanisms, while focusing on the types of biomaterials used in tumor immunotherapy as well as their preparation, processing techniques, principles of action, and recent advancements. Furthermore, this review explores the application potential of biomaterials in tumor immunotherapy, in addition to their underlying challenges.

## Types of tumor immunotherapy

### Cytokines

**Fig. 1:**
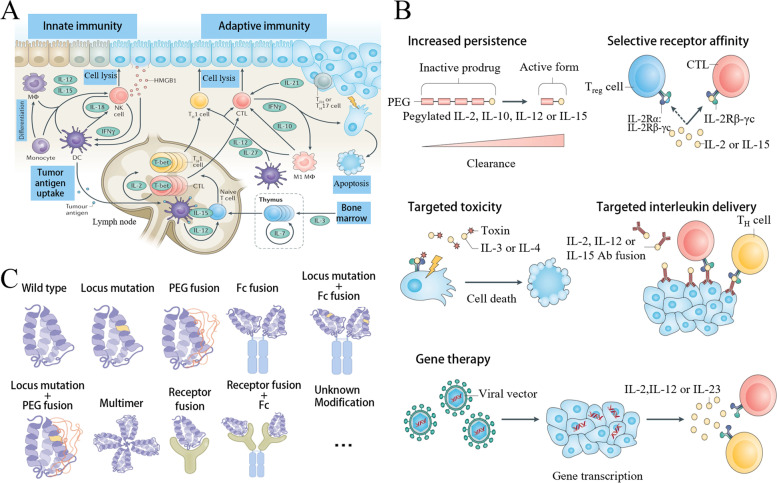
Schematic diagram of cytokine therapy for tumor. **A** Schematic illustration of cancer control mechanisms based on interleukin. (Reproduced with permission from [8] Copyright © 2021, Springer Nature) **B**. Function of recombinant and engineered cytokines. (Reproduced with permission from [8] Copyright © 2021, Springer Nature) **C**. Schematic illustration of super cytokine engineering strategy. (Reproduced with permission from [9] Copyright © 2022, Springer Nature)

Various immune cells (lymphocytes, monocytes, and macrophages) and some non-immune cells (fibroblasts, vascular endothelial cells, and epidermal cells) can secrete cytokines. Cytokines are essentially peptides or glycoproteins, with molecular weights typically below 30 kDa, which provide signals of growth, differentiation, inflammation, or anti-inflammation to various cell types [5]. When cytokines bind to receptors with a high affinity on target cell membranes, these receptors induce intracellular signals that alter gene transcription, followed by the mediation and regulation of inflammatory reaction and immune response, tissue repair, and stimulation of hematopoiesis [6]. Cytokines in cancer immunotherapy act by limiting tumor cell growth either directly (using the anti-proliferative or apoptosis-promoting activities of several cytokines) or indirectly (through the stimulation of cytotoxic activity of immune cells over that of tumor cells) (Fig. 1A) [7].

Cytokines are among the earliest drugs used in tumor immunotherapy. Interferon-α (IFN-α) and interleukin-2 (IL-2), as representative drugs in clinical practice, are used for the treatment of several malignant tumors with the approval of the US Food and Drug Administration (FDA) [7, 10]. Research studies have confirmed the use of IL-2 in treating renal cell carcinoma and advanced metastatic melanoma. Nevertheless, high-dose IL-2 monotherapy exhibits high toxicity, moderate efficacy, and other limitations; thus, it is rarely used alone and has been replaced by other immunotherapeutic agents [11, 12]. IFN-α is primarily applied in treating follicular Hodgkin's lymphoma, hair-cell leukemia, melanoma, and Kaposi's sarcoma [11]. According to statistics, IFN-α demonstrated certain activity in treating low-grade inert non-Hodgkin's lymphoma; however, the remission rate was less than ideal [13-15]. IL-2 at high dose and IFN-α have low response rate and strong toxicity, making them limited to immune checkpoint inhibitors (ICIs) and targeted therapies [16].

With the exception of IFN-α and IL-2, some clinical trials on the immunobiology of other cytokines (such as IL-15, IL-2, IL-10, IL-12, IL-8, TNF-α, and TGF-β) have shown promising results [17]. In recent years, scientists have conducted extensive research to improve the effectiveness of cytokines in cancer treatment. The structural principles and functional signals regarding the interaction of cytokines and receptors have been deeply investigated; for instance, artificially modifying cytokine signal from protein engineering and synthetic immunology has exhibited strong feasibility and effectiveness. This includes recombinant engineered cytokines for increased persistence, targeted toxicity, and gene therapy (Fig. 1B) [8]. Furthermore, there are supercytokines, which are produced through various strategies. Initial frameworks usually consider a natural cytokine (wild-type), using conventional molecular biology techniques for manipulating the intrinsic properties, thereby introducing specific mutations. Pegylation (PEG fusion) can improve the bioavailability and half-life of cytokines, and fusion with targeted specific antibodies (Fc fusion) helps to achieve targeted delivery. Finally, more sophisticated techniques include dimer or poly for the enhancement and synergy of activity, receptor fusion for the moderation of activity, and specific binding antibody fusion for the construction of targeted delivery (Fig. 1C) [9].

### Antibodies

Tumor cell-surface antigens can elude immune surveillance and promoting their proliferation, which is of great significance. Some antigens have a direct effect on tumor onset and growth. Researchers have confirmed that antigens involved in tumor cell growth are clinically effective therapeutic targets and have, therefore, developed their respective antibody drugs [18]. When B cells create monoclonal antibodies, they target certain antigen domains, bind to particular antigens, and prevent the attachment of the antigens to ligands. Drugs that contain monoclonal antibodies are capable of driving T cells to the tumor site, directly targeting the tumor cells, and changing the host response to the tumor, thereby suppressing or completely eradicating the tumor [19]. Monoclonal antibody drugs have enjoyed a broad application in the treatment of various tumors, like B lymphoblastic tumor, Hodgkin's lymphoma, colorectal cancer, breast cancer (BC), gastric cancer, esophageal cancer, and chronic lymphocytic leukemia [18, 20]. Monoclonal antibody-targeted treatment is a research hotspot that depends on its wide application potential. By 2021, the US FDA had approved more than 100 antibody drugs [21]. Antibody immunotherapy involves treating cancer using therapeutic, immunosuppressive myeloid-derived suppressor cells (MDSCs) and regulatory T cells (Tregs), and ICI monoclonal antibodies.

#### Therapeutic monoclonal antibody

**Fig. 2:**
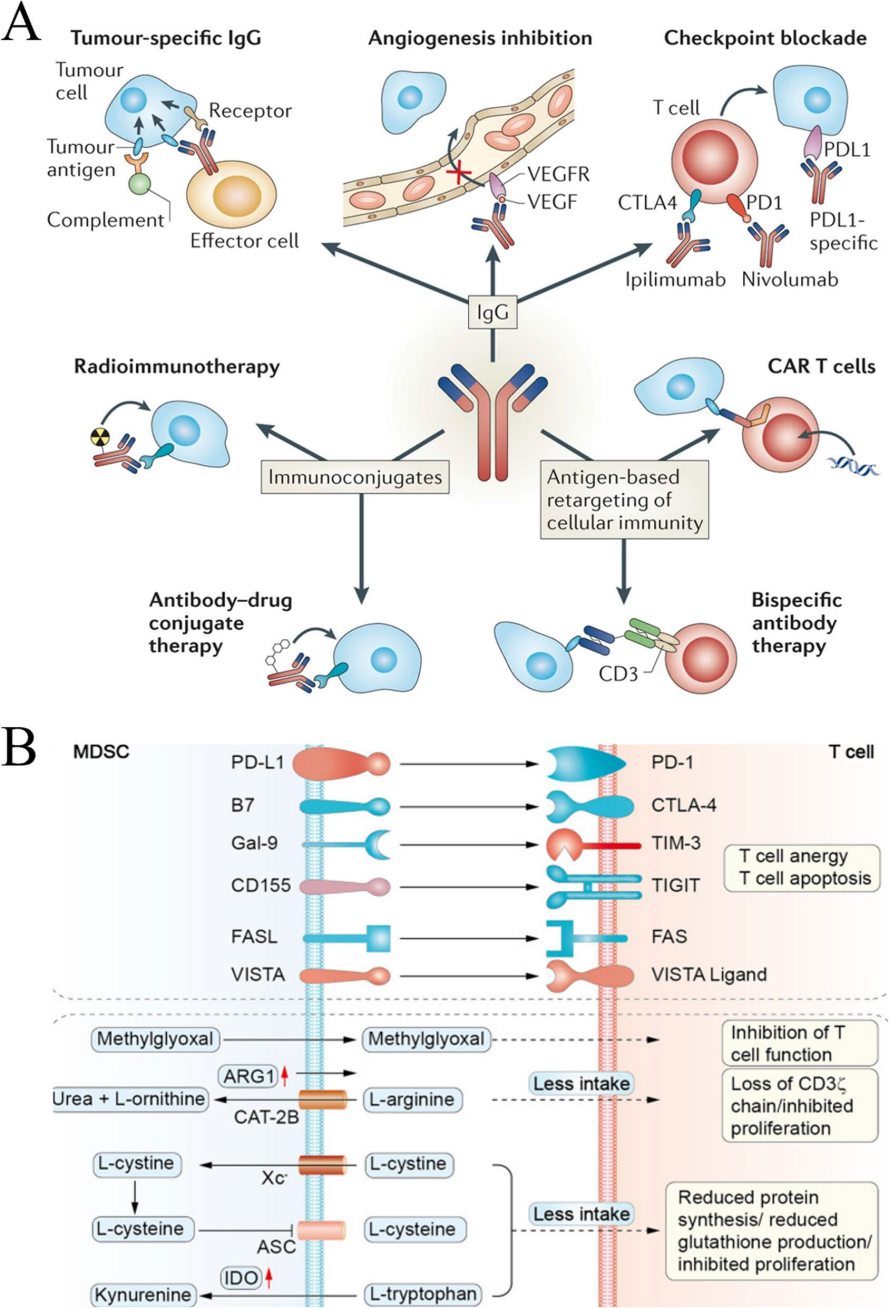
Schematic diagram of antibody therapy for tumor. **A** Schematic illustration of tumor immunotherapy strategies based on monoclonal antibodies. (Reproduced with permission from [22] Copyright © 2015, Springer Nature) **B** Schematic illustration of the mechanism of MDSC-mediated T cell inhibition. (Reproduced with permission from [26]  Copyright © 2021, Springer Nature) [26]

Therapeutic monoclonal antibodies include an antigen-binding segment (Fab segment) used to bind tumor antigens and Fc segment that interacts with Fc receptors on immune cells; they play a therapeutic role mainly through antibody-dependent cell-medicated cytotoxicity and complement effect (Fig. 2A) [22]. The use of rituximab, ofatumumab, and alemtuzumab via this mechanism have all received FDA approval. Second-generation monoclonal antibodies, such as blinatumomab and emicizumab, represented by a bispecific antibody (bsAb) are commercially available [23]. bsAbs have two different antigen-binding sites that can simultaneously attach to two target antigens or two different epitopes of one antigen, thereby contributing to tumor resistance. Compared with ordinary monoclonal antibodies, bsAbs have high specificity, good stability, less dosage requirements, and less adverse reactions; in addition, they provide tremendous benefits in the clinical therapy of tumors [24]. In recent years, researchers have developed innovative bsAbs against triple negative breast cancer (TNBC), which can redirect TNBC immune cells and target key receptors expressed on TNBC cells for effective treatment [25].

While most bi-functional antibodies are developed to allow interaction between immune and tumor cells, there are other therapeutic strategies, including linking cells to a "payload" (for example, a drug) or blocking signaling pathways in the TME, such as the inhibition of cytotoxic T lymphocyte-associated antigen-4 (CTLA-4) and programmed death-1 (PD-1) channels [27]. The combination of multiple monoclonal antibodies may be more effective or less toxic than monotherapy use. For instance, collective use of ipilimumab (targeting PD-1, an immune checkpoint receptor) and nivolumab (targeting CTLA-4, a co-stimulatory receptor) can strengthen the anti-tumor response in the treatment of melanoma [28], advanced renal cell carcinoma [29], and esophageal carcinoma [30]. This combination exhibits greater effectiveness and stronger toxicity compared to the single use of a monoclonal antibody. Pertuzumab and trastuzumab in combination with taxane treat human epidermal growth factor receptor-2-positive BC. Both monoclonal antibodies target th receptor, and the effectiveness and toxicity combined with taxane is more powerful than that of trastuzumab combined with taxane [31].

#### Immunosuppressive MDSCs and Treg monoclonal antibodies

Immunosuppressive cells include type 1 Tregs, CD8^+^ regulatory T cells, type 3 T helper cells, classic CD4^+^CD25^+^FoxP3^+^ regulatory T cells, regulatory B cells, M2 macrophages, regulatory natural killer (NK) cells, tolerant dendritic cells, and MDSCs. After immunosuppressive cells are pulled to the TME, tumor tissues strongly inhibit and suppress the body’s immune cell response and achieve immune evasion (Fig. 2B) [26, 32]. In addition, immunosuppressive cells limit the effectiveness of many tumor treatments. Therefore, their removal can partially improve the body’s immune response to tumors [33]. Based on this, Qin et al. used therapeutic peptides (Pep-H6 and Pep-G3) produced by a competing polypeptide phage platform in an animal model to successfully eliminate MDSCs in the blood, spleen, and tumor, thereby inhibiting tumor growth [33]. In addition, according to other studies, daclizumab and denileukin diftitox can treat metastatic BCs by eliminating Tregs in the tumors [34]. Lu et al. designed an IR-700 dye-coupled photosensitive anti-CD73 antibody to target the elimination of tumor-infiltrating immunosuppressive cells expressing CD73 molecules, which significantly improved the therapeutic resistance of ICIs. Notably, this study achieved the first comprehensive elimination of infiltrating immunosuppressive cells in tumors [35].

#### ICI monoclonal antibodies

ICIs refer to a class of immunosuppressive molecules on immune cells, capable of regulating the extent of immune activation, which is crucial in avoiding autoimmunity. However, tumor cells use immune checkpoints to inhibit the body’s immune response and prevent their removal by the immune system [36, 37]. Hence, antibody drugs corresponding to normal inhibitory immune checkpoints are designed to effectively restore antitumor immunity mediated by T cells [38] and achieve tumor clearance. Ipilimumab was the first ICI authorized by the FDA in 2011; it serves as an antibody against CTLA-4 that can be used to treat advanced melanoma via suppression of the inhibitory signal of CTLA-4 to induce activated CTL [39]. Pembrolizumab was the first PD-1 inhibitor approved by the FDA in 2014, and it is used for treating lung cancer and melanoma [40]. Atezolizumab was the first FDA-approved PD-L1 inhibitor in 2016 that was used for treating bladder cancer [41]. The anti-PD-1 monoclonal antibody sintilimab combined with oral multi-target tyrosine kinase inhibitor anlotinib was recently proven to be effective in treating recurrent or advanced endometrial cancer [42], cervical cancer [43], advanced non-small cell cancer [44], and advanced liver cancer [45]; in addition, the toxicity of this combination is controllable. Similarly, for advanced solid tumors (especially gastric cancer), a new bi-functional fusion protein, SHR-1701 (consisting of PD-L1 monoclonal antibodies fused to the extracellular domain of TGF-β receptor II), was shown to have effective antitumor activity and demonstrate acceptable safety in human trials [46]. As research continues, more checkpoints are being discovered and drugs are being developed to target these checkpoints.

### Adoptive cell therapy (ACT)

**Fig. 3:**
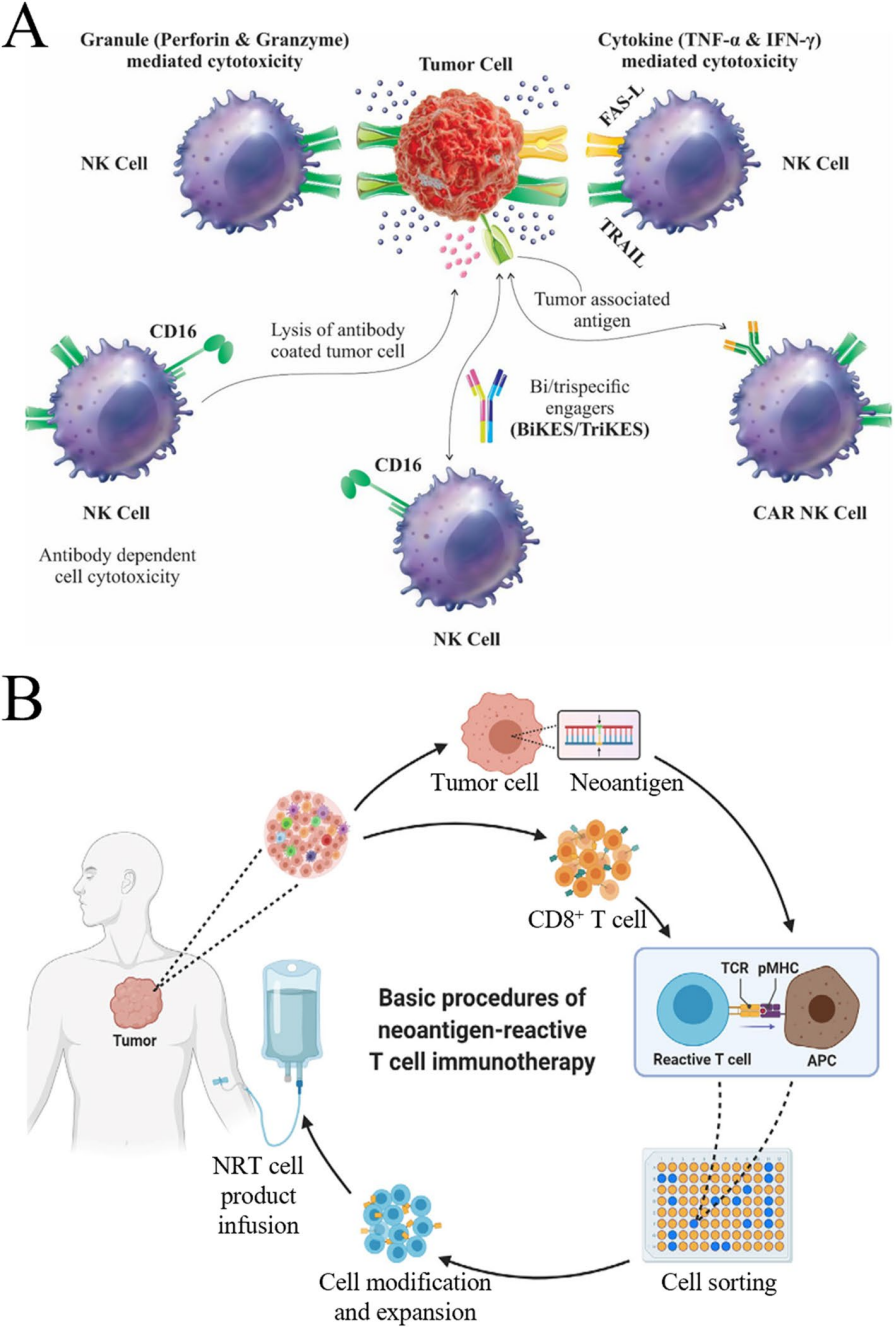
Schematic diagram of ACT for cancer. **A** Mechanisms of NK cell cytotoxicity against tumors. (Reproduced with permission from [47] Copyright © 2021, Frontiers in Oncology) **B**. Schematic diagram of the basic steps of NRT immunotherapy. (Reproduced with permission from [53]  Copyright © 2021, Medcomm) [53]

ACT, belonging to a group of passive immunotherapies, entails removing active immune cells from the patient's body, modifying or activating them in vitro to increase their number by a factor of thousands or to enhance their capacity for targeted killing, and reintroducing immune cells into the body to eradicate blood and tissue pathogens, cancer cells, and mutated cells. ACT can be divided into two categories based on whether the effector cells have foreign gene expression. One category requires genetic engineering, such as chimeric antigen receptor T cell (CAR-T) and T cell receptor chimeric T cell (TCR-T) therapy that genetically modify T cells for the expression of CAR or TCR, respectively; CAR-NK therapy expresses CAR on the NK cell membrane surface (Fig. 3A). The other category refers to the separation and screening of immune cells from peripheral blood or tumors in situ. After expansion and activation in vitro, immune cells are injected back into the patient’s body to receive anti-tumor therapy, like tumor-infiltrating lymphocyte (TIL) therapy, cytokine-induced killing (CIK) cell therapy, lymphokine-activated killing cell therapy, and natural killing cell therapy [47-52]. Neoantigen-reactive T cells (NRTs), which have been studied in recent years, target the antigens expressed by tumor cells and can completely eliminate tumors and retain normal tissues, thereby achieving better anti-tumor effects than the previously mentioned methods (Fig. 3B) [53]. Nevertheless, solid tumors exhibit heterogeneity and rarely express one tumor-specific antigen [54]. The immunosuppressive checkpoint signals within the tumor limit the application of ACT therapy in the treatment of solid tumors. In addition, adoptive immune cells may gradually die soon after being reintroduced into the body and fail to proliferate effectively, resulting in a shorter treatment duration. Adoptive immune cell therapy refers to the process of removing immunosuppressive cells from the body to prolong the survival time of adoptive immune cells via high-dose chemotherapy or radiotherapy. However, the resultant side effects include stomatitis, mucositis, diarrhea, vomiting, and hair loss [55]. To address this obstacle, several experiments have been conducted. Recently, specific stimulation of o9R signaling in TCR-T cells and CAR-T cells using a modified IL-2 cytokine receptor platform has assisted in enhancing the antitumor activity of T cells, even in refractory tumor models with anti-immunotherapy or in harsh environments lacking chemotherapy or radiotherapy [56].

### Tumor vaccines

**Fig. 4:**
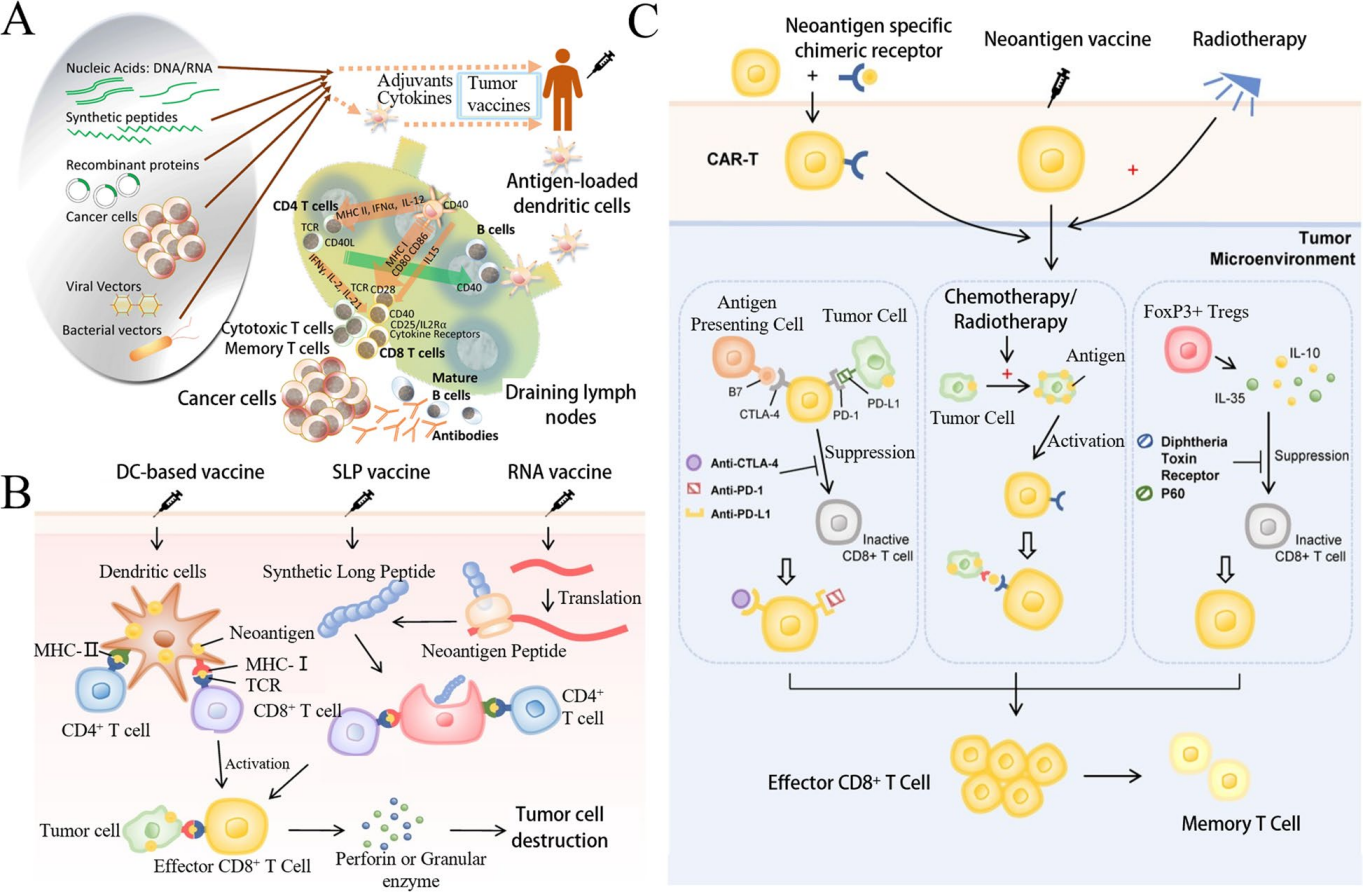
Schematic diagram of tumor vaccine therapy for tumor. **A** Cancer vaccine platforms and interactions in the immune system. (Reproduced with permission from [57] Copyright © 2019, F1000Research) **B.** The main types of neoantigen vaccines. (Reproduced with permission from [62] Copyright © 2019, Molecular Cancer) **C**. Neoantigen vaccines in combination with other therapies (ICI, radiotherapy, chemotherapy, and CAR-T). (Reproduced with permission from [62] Copyright © 2019, Molecular Cancer) [62]

Tumor vaccines, including dendritic cell (DC), gene, polypeptide, and tumor vaccines, activate the body’s immune system to kill tumor cells (Fig. 4A). DC vaccines can obtain the characteristic information of tumor antigens and present it to lymphocytes [57]. In 2010, the FDA approved Provenge, the first DC vaccine, for the treatment of prostate cancer [58]. Gene vaccines encompass the use of genetic engineering technology to encode the gene of foreign antigen, recombine a viral vector or plasmid, and construct a eukaryotic expression vector; this is done by using the synthesis system of host cell proteins to synthesize the foreign antigen protein and stimulate the body to generate an immune response to the antigen, thereby maintaining relative long-term immune response. Currently, all gene vaccines for tumor therapy are in the stage of clinical research [59]. To date, there have been thousands of clinical trials on tumor vaccine therapy, mainly for malignant tumors, such as melanoma, BC, and lung cancer [60]. In 2017, personalized vaccines began to attract attention, which are a selection of appropriate peptide vaccines customized according to the patient’s human leukocyte antigen-A types and pre-existing immune memory to produce more rapid and stronger immune response and achieve personalized therapy (Fig. 4B) [61].

Sneh et al. proposed that the future direction of personalized cancer vaccines was the combination of DNA, RNA, or peptides encoded by target antigens. They are neoantigen-specific and bind to an adjuvant so that they can be delivered by antigen-presenting cells (APCs), and so that specific T cells are activated, clone-amplified, and targeted to specific tumors [63]. Although personalized cancer vaccines are useful, there are some practical limitations. mRNA possesses a large molecular weight and has a negative charge, causing difficulty in penetration of the lipid bilayer on the cell membrane, which also possesses a negative charge. In addition, mRNA is easily degraded by immune cells or enzymatic digestion; therefore, the preparation of personalized cancer vaccines is limited by the selection of an appropriate vaccine delivery system [64]. Usage of neoantigen vaccines in combination with other therapies is a new feasible approach (Fig. 4C). Shou et al. found in clinical trials that neoantigen vaccination following local radiofrequency ablation could enhance the clinical and immune response in patients with various cancer types. Animal studies have explained the synergistic anti-tumor potential of the previously discussed two approaches, which could be more effectively strengthened via immune checkpoint inhibition [62, 65].

### Oncolytic virus (OV) immunotherapy

OV refers to a class of viruses existing in nature or those that can be genetically modified to specifically infect and kill tumor cells. In contrast to gene therapy in which viruses serve as transgene delivery vectors, OVs can essentially be active drugs [66]. Most OVs currently employed in clinical studies are genetically modified (adenovirus, pox virus, herpes simplex virus, and parvovirus) and can eliminate tumor cells through various ways [67]. They can also be used for imaging and antitumor activity. OH2, as a monotherapy or a combined ICI, is applied for tumor localization in patients with advanced solid tumors; in such cases, the reporter gene in the modified OV replicates and emits fluorescence during expression. Using established tumors as the in situ source of neoantigen vaccines, OVs help to regress the distant uninfected tumors via cross-presentation [67]. Furthermore, OH2 refers to an oncolytic herpes simplex virus type 2 generated from genetic engineering. According to clinical trials by Zhang et al., intracellularly injecting OH2 into tumors provides good tolerance and presents a constant and effective tumor resistance for patients who develop metastatic esophageal and rectal cancer [68]. Olvi-Vec is a modified vaccinia virus that can activate oncolytic reaction and immunoreaction. Adhavi et al. carried out clinical trials on patients with platinum-resistant or refractory ovarian cancer and found that the peritoneal Olvi-Vec therapy demonstrated strong safety, clinical activity, and immune activation; these findings are worthy of further research and development [69]. However, recent studies have shown that antigenic peptides can be used as adjuvants with OVs in immunotherapy, which is an important step towards personalized therapy (Table 1) [70].

**Table 1:** Summary of various immunotherapies for tumors

Type of tumor immunotherapies	Principle	Advantages	Limitations
Cytokine	Activating, proliferating, or differentiating anti-tumor immune cells	Wide application, visible curative effects, and few side effects	Long treatment time, toxicity, and limited treatment scope
Immune checkpoint-blocking	Targeting the surface regulatory receptor of T lymphocytes to activate proliferative tumor-specific T lymphocytes	Low toxicity, long-term effects, and excellent potential for combination with traditional tumor-targeting therapy and other immunotherapies	Only releases the binding of T lymphocytes already at the edge of the tumor or enhance presentation, cannot promote T cells to attack the tumor, and limited response rate
Adoptive cell immunotherapy	Adopting tumor-specific T lymphocytes	Good curative effects and specific killing of various tumor cells	Difficulty of separating and expanding tumor-infiltrating lymphocytes, and bottlenecks in industrial production
Tumor vaccine	Present tumor antigen triggering anti-tumor antibody and cellular immunity	Good anti-tumor specificity, immune memory, and good prospects of combined use with immunomodulatory antibodies	High cost, undefined adverse reaction, and no drugs with good efficacy
Oncolytic virus	Proliferation of oncolytic virus inducing tumor lysis	Excellent curative effect on small tumor metastasis and good prospects of combined use with immunomodulatory antibodies	Limited efficacy and inconsistent safety for primary large tumors

### Combination therapy

Considering the complex TME and existence of immune escape, the current single tumor immunotherapy cannot effectively play its role. To solve this problem, researchers have turned their attention to tumor combined immunotherapy. Specifically, preclinical trials have verified that the co-treatment of block PD-1 with CTLA-4, which negatively regulate the activation of T cells, can strengthen anti-tumor immune responses compared to a single drug therapy. This was the first breakthrough in the treatment of metastatic melanoma [71, 72]. Zhen et al. combined the neoantigen DC vaccine with PD-1 antibody to treat advanced non-squamous non-small cell lung cancer (NSCLC) as a first-line treatment, and the results showed good effects. According to the preliminary results, this combination exhibited feasibility, safety, and effectiveness.

Combined immunotherapy targets immune cells and inhibits the immunosuppressive microenvironment, thereby activating the immune system and contributing to its long-term anti-tumor action [73]. However, traditional surgery, radiotherapy, chemotherapy, and immunotherapy have many drawbacks that cannot be determined or avoided at present. For that reason, researchers have focused on the co-treatment of tumor immunotherapy with chemotherapy, targeted therapy, antiangiogenic drugs, and other immunotherapies [74]. To date, the FDA has approved several combination therapies for different cancer types. More combinations of immunotherapy, radiotherapy, chemotherapy, targeted therapy, and immunotherapy are being tested clinically to improve anti-cancer efficacy by targeting multiple defects in the immune cycle and inherent changes in cancer [74].

## Biomaterials used in tumor immunotherapy

Immunotherapy is an effective strategy for cancer treatment, and clinical trials have verified its strong efficacy in clinical practice. However, tumor immunotherapy cannot be properly applied clinically owing to low immunogenicity, limited specificity, low delivery efficiency, and certain adverse side effects. Increasing the dosage can improve efficacy to some extent, but patients may experience higher risk or untargeted side effects [75]. Fortunately, advanced biomaterials have led to new solutions that effectively promote anti-tumor immune responses, improve therapeutic efficacy, target drug delivery, and reduce adverse effects [76, 77]. In the following, we describe three aspects of biomaterials used in tumor immunotherapy: their categories **(**Table 2**)**, preparation and processing techniques, and principles of action.

**Table 2:** Summary of biomaterials for tumor immunotherapy

Nature			Drugs	Preparation method	Therapy or mechanism	Anticancer performance	Refs
Inorganic biomaterials	Metallic oxide	Mn oxide NP	AuNC@MnO_2_(AM)	Galvanic replacement method	PDT, Immunotherapy	Destroyed the tumor directly, inhibited lung metastasis	[78]
			BSA-MnO_2_IPI549(BMI)	Direct mixing method	MRI-visible immunotherapy	Immunosuppressive PD-L1, M1-polarization of TAMs, and activation of tumor-suppressive T-lymphocytes	[79]
		Zn oxide NP	Dox-loaded ZnO NPs	Stirring, centrifuging, washing, freeze-drying	Immunotherapy, Chemotherapy	Killed multidrug resistant cancer cells, downregulated CD44, promoted cancer cell apoptosis, M1-polarization of TAMs	[80]
			ZnO nanowires	Alkaline solution-based method	Immunotherapy	Significantly inhibited tumor growth in vivo	[81]
		Fe oxide NP	FeNP-delivered CpG particles (FeNP/CpG)	Co-precipitation method	Immunotherapy	Inhibited tumor growth and lung metastasis	[82]
			Fe_3_O_4_–OVA NP vaccines	Mild vortexing method	Immunotherapy	Significantly influenced immune response and tumor inhibition	[83]
	Silicon NP		ZnPP@MSN-RGDyK	Step-by-step synthesis	PDT, Immunotherapy	Precisely targeted β3-int to inhibit PD-L1 in an NSCLC-SM mouse model	[84]
			Mesoporous silicon NPs	Stirring, centrifuging	Immunotherapy, Chemotherapy	Inhibited the growth and metastasis of tumor cells	[85]
			Cancer cell membrane coated Fe_3_O_4_@SiO_2_ magnetic NPs	Hydrothermal method	Immunotherapy	Activated NK cells, killed tumor cells in vitro	[86]
			Black porous silicon NPs	Cocktail regimen	PTT, Immunotherapy	Inhibited distant and metastatic tumors	[87]
			TME—regulating SiO_2_@MnO_2_ NPs (SM NPs)	Hydrothermal method	Radiotherapy (RT), Immunotherapy	Inhibited tumor growth and metastasis	[88]
	Carbon nanotube (CNT)		Durvalumab/CNT/PEI/aptamer-siRNA chimera (chimera/Durmab/CNT)	Washing, stirring, centrifuging	Immunotherapy	Targeted to bind with HCC cells and silence the Trem2 gene, promoted apoptosis of HepG2 cells, inhibited growth of transplanted tumor	[89]
			OVA-loaded Man-MWCNTs	Physical adsorption	Immunotherapy	Efficiently enhanced DC maturation and cytokine secretion	[90]
			MHR-CpG	Vortexing, incubating	Immunotherapy	Inhibited the growth of prostate cancer cells	[91]
Organic biomaterials	Synthetic polymer materials	PLA	Anti-neu/anti-CD40-NP	Washing, incubating, centrifuging, blocking, resuspending	Immunotherapy	Recognized RNEU tumors and activated DCs	[92]
			LD-indolicidin-PEG-PLA	O/W emulsion method	Immunotherapy	Elicited a T helper (Th)1-dominant antigen-specific immune response and antitumor ability	[93]
			R848-loaded NP	Microwave-assisted ring opening polymerization	Immunotherapy	Efficiently stimulated macrophages	[94]
		PLGA	PLGA-ICG-R837 NPs	O/W single-emulsion method	PTT, Immunotherapy	Eliminated primary tumors, attacked and killed spreading metastatic tumors, and offered immune-memory protection to prevent tumor relapse	[95]
			DOX/aNLG919-loaded CaCO_3_ NPs (DNCaNPs)	Classical double emulsion method	Immunotherapy, Chemotherapy	Inhibited tumor growth in both subcutaneous CT26 colon tumors and orthotopic 4T1 breast tumors	[96]
			Riboxxim/OVA-loaded PLGA NPs	Double emulsification solvent evaporation method	Immunotherapy, targeted therapy	Increased DC maturation and type I IFN cytokine secretion, improved priming and activation of CD8^+^ T cells, inhibited tumor metastasis	[97]
			ONP-302	Emulsion mixing method	Immunotherapy	Activated STING/IL-15/NK cell mechanism reprogrammed myeloid cells, increased expression of PD-1/PD-L1 in TME	[98]
		PEG	CDN-PEG-lipids	Alcohol dissolution method	Immunotherapy	Triggered acute tumor necrosis, and activated DCs and CD8^+^ T cells	[99]
			PEG-SAB-Lip	Ethanol injection method	Immunotherapy, Chemotherapy	Inactivated tumor-associated fibroblasts, and inhibited tumor growth and lung metastasis	[100]
			Cascaded pH-activated supramolecular nanoprodrug (PDNP)	Two‐kind aminaldehyde reaction	Immunotherapy, Chemotherapy	Facilitated DC maturation and CD8^+^ T cell infiltration, primed CTL proliferation, and boosted antitumor immune cycle	[101]
			PBE-modified TRP2 nanovaccine	Dialysis method	Immunotherapy	Promoted cellular uptake, stimulated DC maturation, enhanced lymph node retention, and improved T cell activation	[102]
			DMP@NPs	Washing, stirring, centrifuging	Immunotherapy	Inhibited tumor growth in colorectal cancer	[103]
			TA-CA-DOX-PEG	Amidation reaction	Immunotherapy, Chemotherapy	Increased CD8^+^ T cells, and mature DCs, decreased Treg infiltration	[104]
		PEI	PEI-EGFR-PD-L1-siRNA dual functional nano-vaccine	Reverse emulsification method	Immunotherapy, Gene therapy	Inhibited the expression of PD-L1, regulated immunosuppressive TME	[105]
			PEI-OA MNP	Thin-film hydration method	Immunotherapy, Chemotherapy	Increased tumor invasion and activation of CD8^+^ T cells	[106]
			PVA-PEI-Mg^2+^ scaffold hydrogel (PEIGel)	Stirring, mixing	Immunotherapy	Eliminated primary tumors and remote metastases and prevented tumor relapse after surgical resection	[107]
	Natural biomacromolecular materials	Polysaccharides	Alginate/κ-carrageenan oral microcapsules loaded with Agaricus bisporus polysaccharides MH751906	Stirring, washing, drying	Immunotherapy	Regulated both BCL2 and TGF surviving genes and upregulated IkappaB-α gene expression to inhibit colon cancer cells	[108]
			PEG-HA-OVA/PPLs	-	Immunotherapy	Inhibited the expression of PD-L1 in CD44^+^ tumor cells and CD44^+^ DCs, and reprogrammed TME	[109]
			OPBP-1-loaded trimethyl chitosan hydrogel	2-step methylation method	Immunotherapy	Inhibited tumor growth and enhanced infiltration and function of CD8^+^ T cells	[110]
			NOCC-CpG/OX-M, Ncom Gel	Dissolving, mixing, cross-linking, freeze-drying	Immunotherapy	Enhanced antigen presentation to T cells and showed significant antitumor response in a melanoma model	[111]
		Silk fibroin	Dinutuximab-loaded lyophilized SF foams	Mixing, freezing, lyophilizing, annealing	Immunotherapy	Inhibited tumor growth in orthotopic neuroblastoma model	[112]
			CCs-SF / DOX	Degumming, dissolving, filtering, freeze-drying, mineralizing	Immunotherapy, Chemotherapy	Promotes M1-like macrophage polarization, reversed immunosuppressive TME	[113]
			SH@FP@CaCO_3_	Stirring, mixing, mineralizing	Immunotherapy	Enhanced immunogenicity and reversed immunosuppression in TME	[114]
			Antigen/Adjuvant-loaded macroporous SF microspheres	Ice templating self-assembly technique	Immunotherapy	Suppressed tumor growth by improving CTL response	[115]
		Gelatin	CRAd-IL12-IL15 + CIKs/gelatin	Dissolving, stirring, dialyzing, lyophilizing	Immunotherapy	Sustained to induce potent anti-tumor immune responses with single administration	[116]
			Micro-hydrogel injectables	Two-step process, pipette tip-based microfluidic device	Immunotherapy	Served as carrier systems to augment CAR-T treatment of solid tumors	[117]
			STB-ICI	Dissolving, stirring, mixing	Immunotherapy	Reduced tumor growth, increased CD8 T cell level, and induced increased levels of tumor-infiltrating CD4 helper T cells, CD8 cytotoxic T cells, and tumor death	[118]
	Cell-derived bioactive materials	RBC membrane	BPQD-RMNV	Extrusion method	Immunotherapy, PTT	Inhibited primary and secondary TNBC cell growth	[119]
			Fe_3_O_4_-ICG@IRM NPs	Ultrasonic fusion method	Immunotherapy, PTT	Inhibited the growth of primary and metastatic ovarian cancer cells	[120]
		Platelet membrane	PNP-R848	Single emulsion process	Immunotherapy	Inhibited the growth of colorectal cancer and BC cells and inhibited lung metastasis	[121]
			PCDD NPs	Gradient centrifugation method	Immunotherapy, Chemotherapy, PDT	Inhibited the growth of primary and distant melanoma and lung metastasis	[122]
		Macrophage cell membrane	I-P@NPs@M	Ultrasound emulsification method	Immunotherapy, Chemotherapy, PDT	Inhibited BC cell growth and lung metastasis	[123]
			NPR@TAMM	Separation coating method	Immunotherapy, PDT	Inhibited tumor growth and metastasis	[124]
		Stem cell membrane	PDA-DOX/siPD-L1@SCM	Stirrig, mixing, centrifuging, washing	Immunotherapy, Chemotherapy, Gene therapy	Inhibited prostate cancer growth and bone metastasis	[125]
			HMnO2-TAT@PTX NPs	Three-step reaction	Immunotherapy, Chemotherapy	Inhibited NSCLC growth, metastasis and recurrence	[126]
		Tumor cell membrane	C1498-mOVA-AMCNPs	Overlap PCR	Immunotherapy	Activated AML-specific immune responses and provided long-term anti-leukemic survival benefit	[127]
			PEG-NPs	Desalting column purification	Immunotherapy	Elicited strong CTL responses with potent anti-tumor efficacy to inhibit melanoma growth	[128]
		OMV	Engineered OMV-PD1	Separation and purification	Immunotherapy	Regulated the TME to increased anti-tumor efficacy	[129]
			mTOMV	Vesicular hybridization	Immunotherapy	Inhibited tumor growth and lung metastasis	[130]

*MRI* Magnetic resonance imaging, *NP* Nanoparticle, *PDT* Photodynamic therapy, *PTT* Photothermal therapy, *RBC* Red blood cell, *Th* T helper; *NSCLC* Non-small cell lung cancer

### Categories

#### Inorganic materials

##### Metal oxide nanomaterials

Nanomaterials refer to materials with no less than one dimension in a 3D space, with a length of 1–100 nm [131]. Nanomaterials are categorized into metals, metal oxides, polymers, and composite nanomaterials based on their chemical element composition [131]. Metal oxide nanomaterials refer to those formed by the chemical bond between a metal and oxygen elements. Manganese oxide, magnesium oxide, titanium oxide, iron oxide, zinc oxide, cerium oxide, aluminum oxide, and zirconium oxide are all metal oxide nanomaterials. There are various metal oxide nanomaterials with differing properties, which explains their wide application in various fields [132]. Their extremely small size contributes to their special physical and chemical properties (quantum effect, small size effect, and interface effect) [133].

**Fig. 5:**
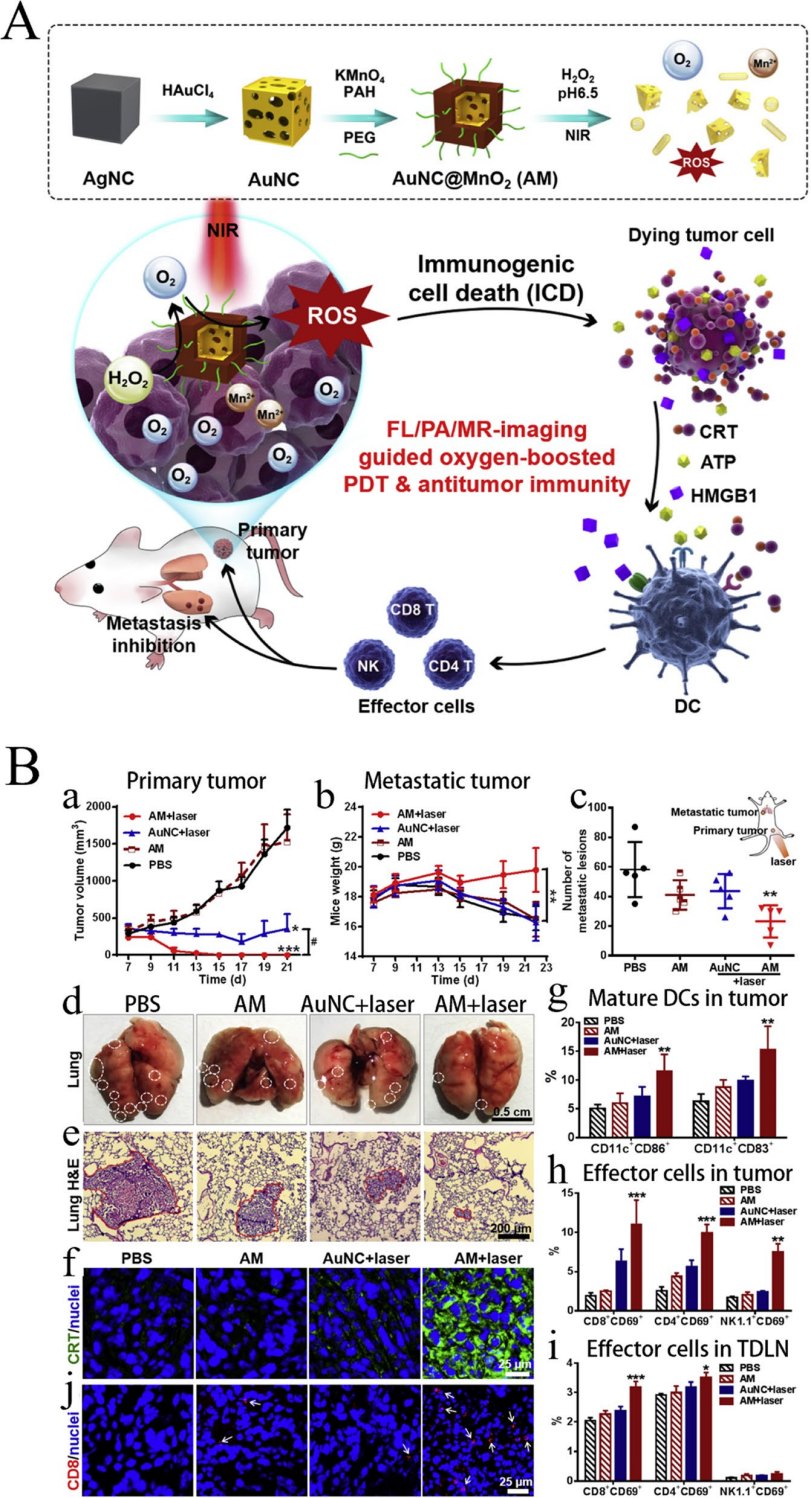
Schematic diagram of manganese based biomaterials for tumor immunotherapy. **A** Schematic illustration of the design and function of AuNC@MnO2 (AM). **B** Oxygen-boosted immunogenic PDT induced antitumor immune response and tumor regression. (a) Tumor growth curves. (b) Mice weight of lung metastasis experiment. (c) The number of metastatic lesions counted from excised lungs. (d) Experimental illustration of anti-metastasis from local PDT. (e) Histological examination of the metastatic nodules in the lung tissue sections. (f) Representative immunofluorescence images of tumor sections stained with specific antibodies against CRT. (g) The expressions of CD86 and CD83 on DCs in tumors at 48 h after treatment. (h-i) The percentage of activated CD8 T cells, CD4 T cells and NK cells in tumors (h) and TDLNs (i) analyzed with flow cytometry at 48 h after treatment. (Reproduced with permission from [78] Copyright © 2017, Elsevier)

**Fig. 6:**
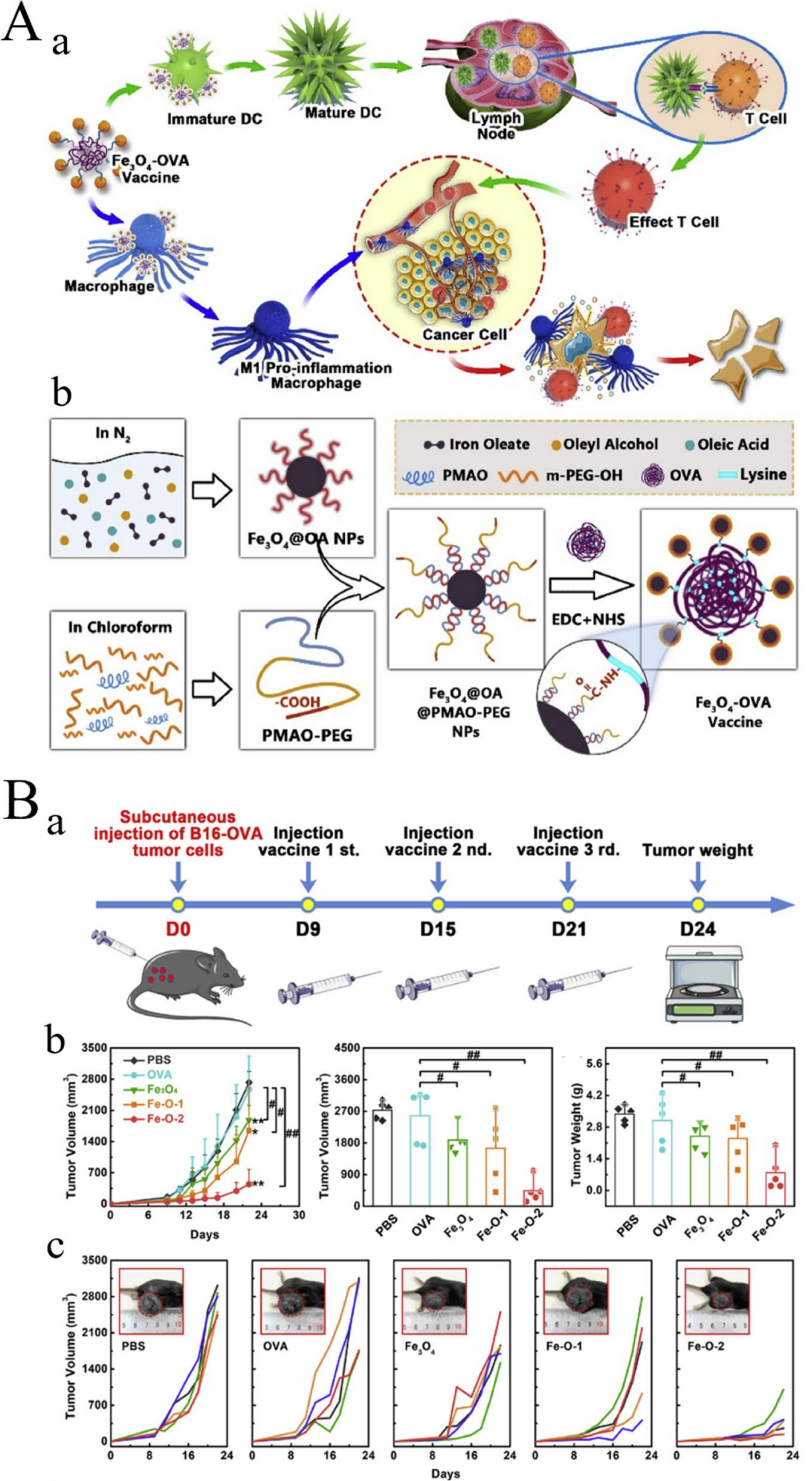
Schematic diagram of iron based biomaterials for tumor immunotherapy. **A** Fe3O4 NPs and Fe3O4OVA nanocomposite. (a) Fe3O4 OVA vaccine strategy schematic. (b) Schematic diagram of Fe3O4 OVA vaccine synthesis. **B** Vaccines for the treatment and prevention of subcutaneous B16-OVA tumors. (a) Schematic diagram of the subcutaneous B16-OVA tumor suppression experimental process. (b) Average tumor growth kinetics, volume and weight at 24 days in the control group and the treatment group. (c) Individual tumor growth kinetics for 24 days. (Reproduced with permission from [154] Copyright © 2019, Elsevier)

1. *Manganese dioxide (MnO*_*2*_*) nanomaterial:* Manganese is an essential trace element of the human body. The body can effectively control its metabolism and demonstrate a certain biocompatibility [134]. Manganese dioxide can decompose rapidly in an acidic environment and can be used as a TME-responsive drug carrier, which makes it specific to tumor tissues so that it does not affect normal tissues [135, 136]. MnO_2_ nanomaterials are capable of catalyzing hydrogen peroxide for oxygen generation, leading to the oxidation of glutathione to glutathione oxide. MnO_2_-based nanomaterials show excellent immunosuppressive TME-regulating abilities by overcoming tumor hypoxia [137-139]. Based on this, Cai et al. prepared AuNC@MnO_2_ (AM) nanoparticles (NPs) as reactive oxygen species (ROS) generators in TME response to O_2_ producers and near infrared triggers for O_2_-sensitized immunogenic photodynamic therapy (PDT) (Fig. 5A, B) [78]. Hypoxia-inducible factor-1α binds to the hypoxia response element in PD-L1 proximal promoter, causing selective upregulation of PD-L1 and inhibiting the antitumor immune response. Therefore, as an O_2_ supplier, MnO_2_ nanomaterials overcome tumor hypoxia by reducing PD-L1 expression, thereby promoting immunotherapy [140]. Based on this, Meng et al. prepared a multifunctional nanomodulator containing MnO_2_-TME response TIME nanomodulator BSA-MnO_2_IPI549, which could regulate the hypoxia state of TME and inhibit MDSC PI3Kγ. IPI549 was released for the simultaneous downregulation of PD-L1 expression and polarization of tumor-associated macrophage (TAM), effectively contributing to tumor immunotherapy [79]. Other MnO_2_ nanomaterials can promote tumor immunotherapy by reprogramming TAMs [141], inhibiting tumor angiogenesis [142], depleting lactate [143], and activating the stimulator of interferon genes (STING) pathway [144] with great potential [145].

2) *ZnO nanomaterials:* Zinc oxide NPs (ZnO NPs) can target cancer cells, enhance cytotoxicity and cell death, and assist in the development of new anti-tumor immunotherapies [146]. Moreover, they do not show any type of hepatotoxicity or nephrotoxicity when used as anticancer agents [147]. Studies have investigated ZnO NPs as nanocarriers for the delivery of different substances, such as drugs, genes, imaging agents, and proteins [148]. Relying on their dissolvability at low pH levels, ZnO NPs serve as appropriate pH-sensitive nanocarriers for delivering tumor-targeted drugs and releasing intracellular drugs [148, 149]. Wang et al. demonstrated that ZnO NPs can not only kill cancer cells but also show synergistic anticancer activity with doxorubicin (DOX). They cause effective downregulation of CD44 expression in cancer (stem-like) cells for the inhibition of cell adhesion, migration, and tumorigenesis. In addition, they can polarize macrophages towards M1-like phenotypes and improve the immunogenicity and anticancer activity of DOX [80]. Sharma et al. synthesized ZnO nanowires radially grown on poly-lactic acid (PLLA) microfibers that possessed a special 3D structure, which were adopted as a therapeutic cancer vaccine. The inorganic-organic hybrid nanocomposite exhibited slight cytotoxicity but could effectively deliver tumor antigens to DCs, which proved that the hybrid nanocomposite could successfully induce tumor antigen-specific cellular immunity and inhibit tumor growth in vivo. Compared with mice immunized with antigen-coated PLLA fibers, ZnO nanowires on PLLA fibers systematically decreased immunosuppressive Tregs and caused the infiltration of more T cells into tumor tissues [81]. Nabil et al. found that ZnO NPs and sorafenib acted synergistically as combination therapy, with better anti-tumor effect and higher safety compared with monotherapy [150]. Therefore, ZnO NPs are a type of multifunctional and multi-target nanocarrier and drug, which have a profound impact on cancer immunotherapy.
3) *Ferric oxide nanomaterials:* Owing to the ability of ferric oxide NPs to track TAMs in vivo, several ferric oxide NPs are used as diagnostic agents in cancer immunotherapy [151, 152]. In addition, they may also have a tumor therapeutic effect because of their ability to alter macrophage polarization. Zhang et al. focused on loading synthetic oligonucleotide guanine cytosine phosphate (CpG) into iron oxide NPs, followed by intratumoral injection into a xenogenic BC model. CpG caused elevated lymphocyte infiltration in tumors, and declined tumor growth and metastasis inhibition in mice [82]. In addition, ferric oxide NP-treated macrophages had an antitumor effect on mouse mammary tumor cell lines in a cell-contact-independent manner, inducing active caspase and inhibiting cell proliferation [153]. Yi et al. found that coupling ovalbumin (OVA) with magnetic iron oxide NPs was sufficient to induce effective DC and macrophage activation and reduce the tumor load of OVA-expressing CT26 in vivo [83]. Luo et al. subsequently found that OVA-coated iron oxide NPs effectively prevented lung metastasis of OVA-expressing cells (Fig. 6A, B) [154]. More recently, Cheng et al. determined the ability of iron oxide NPs to activate tumor immune response after intratumor delivery [155], confirming the finding that they act as immunogenic agents that can lead to the direct activation of innate immune cells and immune response, as well as the indirect stimulation of tumor-specific immune response [156]. Ferric oxide NPs hold great significance in tumor immunotherapy, hence will be further explored and developed.

##### Silicon nanomaterials

Silicon-based nanomaterials (fluorescent silicon NPs [157, 158], silicon nanowires [159], and fluorescent silicon nanorods [160]) have been extensively applied in the diagnosis and treatment of tumors, relying on excellent biological safety and unique optical, electrical, and mechanical properties. Guo et al. used fluorescent silicon NPs to simultaneously load chemotherapy drug DOX and small interfering RNA (siRNA) to construct a bi-functional silicon nanotherapy system, combining biological image-guided chemotherapy with gene therapy for cancer treatment [161]. However, this silicon nanomaterials-based multifunctional treatment system requires complicated post-processing and surface modification, which limits its wide application in the biomedical field to a certain extent [162]. A mesoporous silicon nanodrug delivery system possesses strong biocompatibility and safety, and high voids. It can properly load the drug and supports surface modification; thus, it can be used as a multifunctional carrier of various immune therapeutic agents [163].

**Fig. 7:**
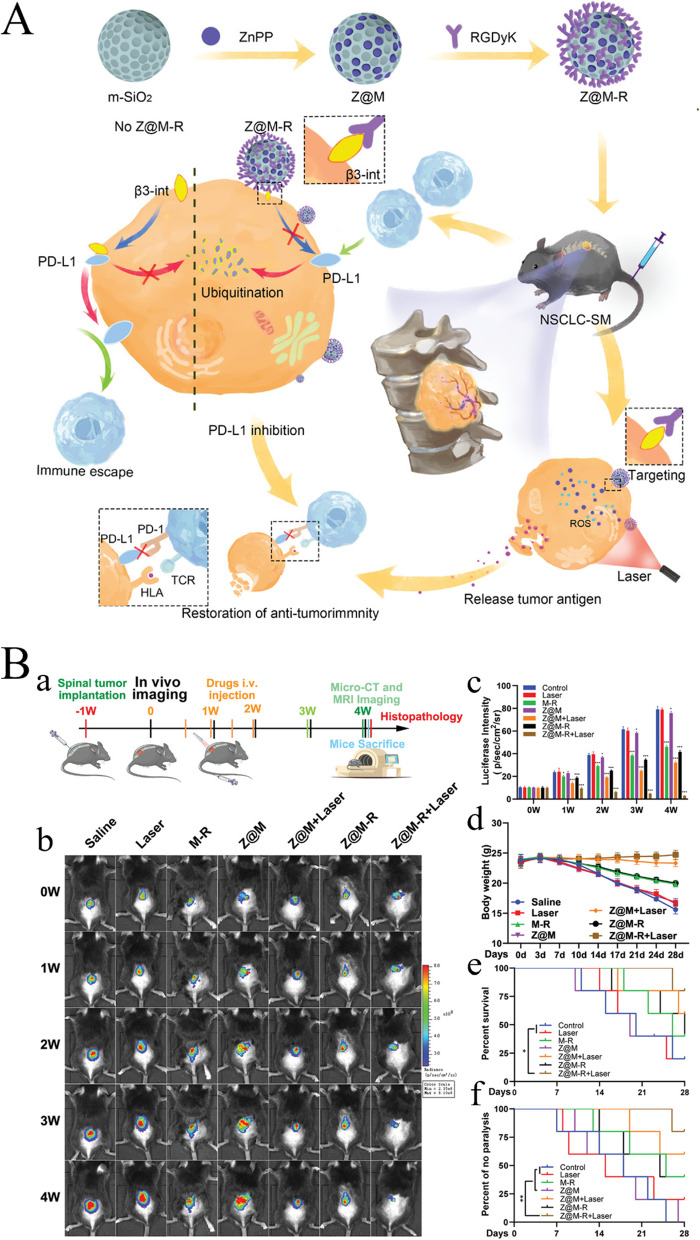
Schematic diagram of silicon NPs based biomaterials for tumor immunotherapy. **A** Schematic illustration of the preparation of ZnPP@MSN-RGDyK or Z@M-R and their immunotherapy NSCLC-SM. **B** Therapeutic effect of Z@M-R NPs on the NSCLC-SM. (a) Schedule of NSCLC-SM treatment. (b) Bioluminescence images and (c) statistical analysis of tumorigenesis. (d) Weight change curve. (e) Survival curve. (f) Paralysis curve. (Reproduced with permission from [84] Copyright © 2022, Wiley‐VCH GmbH)

Kim et al. constructed a ‘nanoparticle loaded nanoparticle’ tumor osmotic drug delivery system based on mesoporous silicon [164], which releases anti-tumor drugs to remove tumor cells in lactic acid (pH = 6.5) and an acidic environment (pH = 5.0) several times. This novel programmed-release mechanism of pH response effectively accounts for the needs of a nano-loaded drug system to extend blood circulation time and enhance the depth of tumor penetration, thus improving the therapeutic effects for cancer. Zhou et al. prepared zinc protoporphyrin-supported mesoporous silicon modified with integrin β3 inhibitor (ZnPP@MSN-RGDyK). This ZnPP@MSN-RGDyK accurately target β3 inhibit PD-L1, showing strong photolysis efficiency and favorable immunotherapy effects in the NSCLC-SM mouse model (Fig. 7A, B) [84]. Wen et al. constructed a universal drug delivery system NP platform based on mesoporous Si NPs and used it to deliver two drugs: DOX and RRx-001. This system could efficiently and accurately target tumor tissues, release drugs through reductive reactions in the TME, and significantly inhibit tumor cell growth and metastasis [85].

Besides mesoporous silica NPs, other silica materials can also be used in tumor immunotherapy. Dan et al. prepared magnetic NPs coated with cell membranes to activate NK cells and enhance anti-tumor effects. Magnetic NPs were coated with silicon dioxide (SiO) and mixed with cancer cell membrane FeO@SiO to construct cancer cell membrane coated with FeO@SiO magnetic NPs. Functionalized NPs that transport cancer-specific antigens can strengthen the expression of activated receptors on the surface and enhance anti-tumor function by secreting soluble cytotoxic effectors, thereby effectively stimulating NK cells [86]. Xu et al. developed black-pore silicon NPs as a photothermal agent and adjuvant for anti-tumor immunotherapy based on photothermal therapy (PTT); combined with DOX and CpG, they helped to inhibit tumor growth and reduced side effects [87]. Yu et al. used TME regulation SiO_2_@MnO to prepare NPs for combined radiotherapy and immunotherapy, demonstrating their regulatory effect on TME and immune response, and inhibitory effect on tumor growth and metastasis [88]. Silicon NPs have great potential in drug delivery and immune response in tumor therapy, with promise of even greater possibilities in the future.

##### Carbon nanotubes (CNTs)

A CNT refers to a synthetic carbon allotrope composed of one (single-walled), two (double-walled), or multiple (multi-walled) graphene layers with concentric rolling. Nanotubes have different morphological characteristics under different formation conditions and methods, having a width within 0.5 to tens of nanometers (decided by the concentric layer number) and a length reaching centimeters [165]. The cylindrical shape and chemical structure (honeycomb carbon lattice) of a CNT confer the material with the potential to transport drugs in organisms [166, 167]. CNTs form a new carrier system that can specifically deliver therapeutic drugs to achieve targeted therapy. They can deliver many peptides, nucleic acids, and drugs with small molecular weight to living cells [168]. The peptide-modified single-walled CNTs exhibit stronger antitumor activity and better tumor-targeting ability [169]. CNTs allow selective and specific destruction of tumor cells by virtue of their tumor-targeting effect combined with magnetic field effect. Many drugs face difficulty in crossing the blood–brain barrier, leading to significant challenges in drug delivery to the brain. Functionalized CNTs are the most appropriate nanocarriers for the brain owing to their ability to cross the biological barrier via endocytosis and passive processes [170]. Additionally, drug-encapsulated functionalized CNTs can cross cell membranes and enter cells through endocytosis or passive diffusion, followed by internalization in organelles and nuclei, and removal from cells through exocytosis and enzymatic degradation [171]. Soluble functionalized CNTs are non-toxic and most are metabolized and eliminated within 24 h of intravenous injection [172].

Recently, Qiang et al. utilized the good tissue penetration capability of CNTs to build Durvalumab/CNT/PEI/aptamer, a siRNA chimera for liver cancer immunotherapy. In vivo and in vitro experiments showed that the chimera could effectively treat liver cancer by specifically combining with the cancer cells and inhibiting the triggering receptor expressed on myeloid cells-2 (Trem2), while having no impact on Trem2 expression in normal tissues [89]. Dong et al. adopted multi-walled CNTs (MWCNTs) under the modification of mannose for the assembly of a specific DC target delivery system. OVA, as a model antigen, could be adsorbed on the surface of mannose-MWCNTs, and the drug loading capacity was strong. The nanotube-antigen complex exhibited weak cytotoxicity to DCs and was effectively phagocytic for DCs to induce DC maturation and in vitro cytokine release [90]. Xia et al. proposed the use of oligodeoxynucleotides containing unmethylated CpG motifs as a nano-delivery system based on MWCNTs conjugated with H3R6 polypeptide (MHR-CpG) for the immunotherapy of prostate cancer, which demonstrated high biocompatibility and immunogenicity. This system could inhibit tumor growth [91]. Jin et al. found that CNT-coupled CpG complex, in addition to showing significant anti-tumor effects in glioma, could also effectively treat local colon tumors and liver metastases, thus showing promise in related target therapy [173]. In addition to anti-tumor effects, CNTs can be used to evaluate the efficacy of tumor immunotherapies. In the experiment by Du et al., electrochemical biosensors on the basis of molybdenum disulfide (MoS_2_) and MWCNTs-modified electrodes and conjugated PD-L1 antibody quantum dots were established as electrochemical and optical markers. The product could detect the difference in tumor cell types, mouse tumors or human cancer cells, in terms of the PD-L1 level, as well as dynamically confirm the biomarkers [174].

#### Organic biomaterials

##### Synthetic polymer materials

Polymer materials are high-molecular weight compounds consisting of many identical, simple structural units repeatedly joined by covalent bonds. Functional polymer materials can be used to produce a variety of polymer nanomaterials for use in biomedical and other fields. As a small-size carrier, polymer NPs can simultaneously transport various hydrophilic/hydrophobic drugs and monoclonal proteins and flexibly adjust their proportions to maximize their synergistic effect [175-177]. The main polymer materials used in tumor immunotherapy are polylactic acid (PLA), polylactic acid-glycolic acid (PLGA), polyethylene glycol (PEG), and polyethylenimine (PEI).

**Fig. 8:**
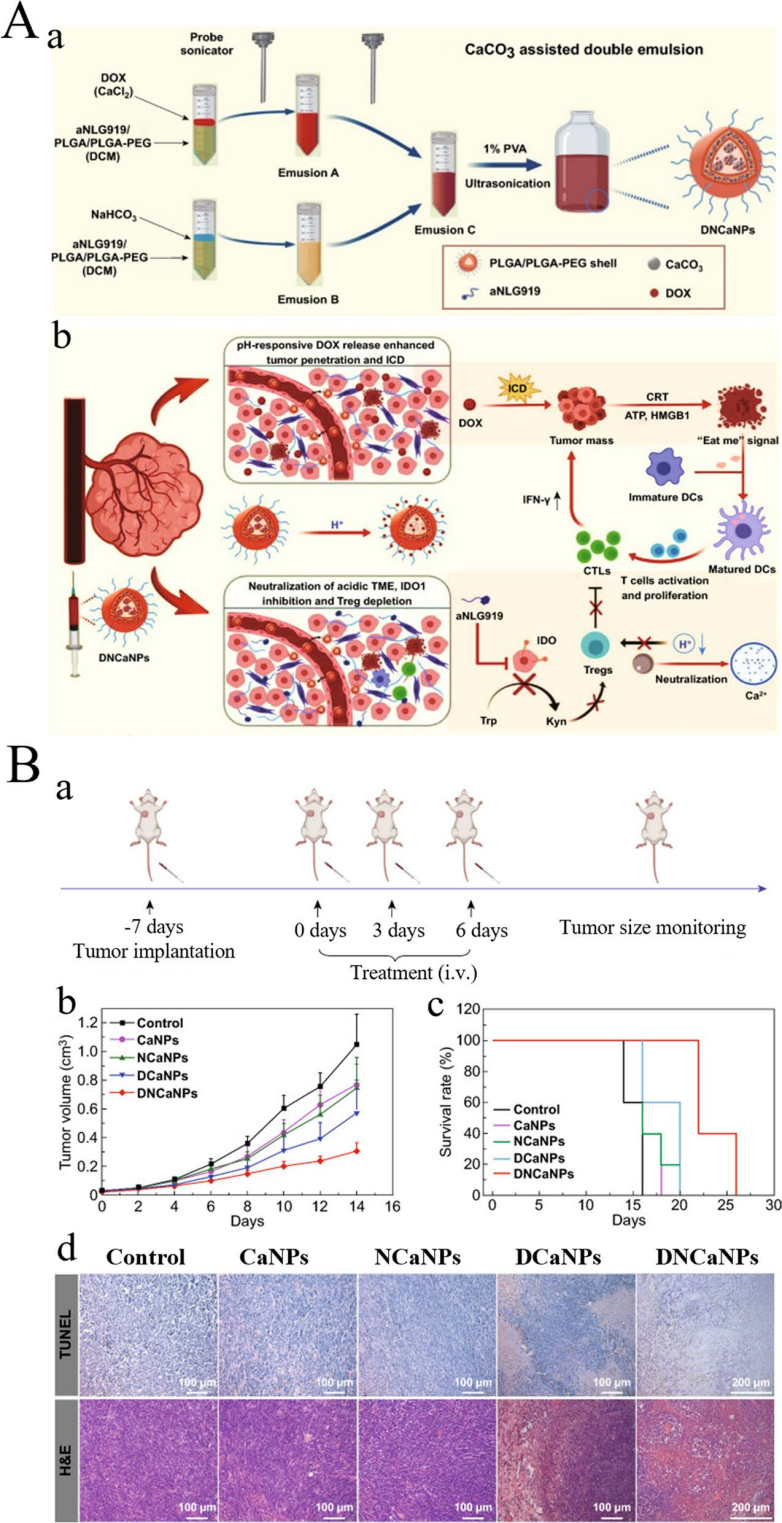
Schematic diagram of PLGA based biomaterials for tumor immunotherapy. **A** Schematic illustration of the (a) preparation process and (b) action step of DNCaNPs for effective cancer chemo- & immunotherapy. **B** In vivo antitumor efficacy of the DNCaNP-mediated chemo- & immunotherapy on orthotopic 4T1 tumor models. (a) The therapeutic schedule. (b) Tumor growth curve and (c) corresponding survival curves of these 4T1 tumors with various treatments. (d) TUNEL and H&E staining of tumor slices collected from the mice with various treatments at day 7. (Reproduced with permission from [96] Copyright © 2020, Nano-Micro Letters)
  PEG units give it "stealth" properties and ensure adequate tumor accumulation. Acidic extracellular pH triggers a dramatic change in particle size and separation of PEG shielding to achieve deep penetration within the tumor. After endocytosis, more acidic endolysosomes take charge of initiating the second stage of size conversion, and the PDNP is broken down into ultra-small substances for ensuring appropriate intracellular delivery. Cascade pH activation of PDNPs effectively induces apoptosis mediated by glutathione E, thereby enhancing the immune response. PDNP binds to anti-PD-1 antibodies to enhance tumor inhibition and prolong mouse survival, suggesting that PDNP is a useful immune adjuvant, contributing to effective immune checkpoint blocking therapy (Fig. 9A, B) [101]. Wang et al. constructed a PEG-modified tyrosinase-related protein 2 (TRP2) nanovaccine containing TRP2 peptide tumor antigen and a diblock copolymer PEG-b-PAsp grafted with phenylborate. They demonstrated that the TRP2 nanovaccine, which relied on elevated negative charge, ROS response, and pH response, was capable of effectively capturing lymph nodes being absorbed by DCs as well as inducing DC maturation. The TRP2 peptide-loaded vector facilitated the strongest T-cell immune response against the melanoma and improved the antitumor efficacy in terms of tumor prevention and treatment without an exogenous adjuvant. Besides, TRP2 nanovaccines restrained tumor growth and prolonged animal survival, which was likely owed to the inhibitory impact on tumor immunosuppression alongside the positive impact on CTL response. The nanovaccine could also safely and reliably deliver other antigens in cancer immunotherapy [102] (Fig. 10)

1. *PLA:* PLA refers to a type of thermoplastic aliphatic polyester, with the monomer of lactic acid. It features nontoxicity, excellent biocompatibility, strong mechanical properties, transparency, and heat resistance, and has enjoyed extensive applications in the fields of food, agriculture and forestry environmental protection, textile, and 3D printing materials [178-181]. In the biomedical field, PLA is used as a drug transporter, tissue engineering scaffold, and bone repair material [182]. PLA is a promising polymer for drug delivery systems in tumor immunotherapy. Dominguez et al. prepared PLA NPs with covalent conjugation using anti-RNEU and anti-CD40 antibodies to induce an anti-tumor immune response, thereby completely eliminating tumors and allowing protective memory response. Further, the NPs initiated specific activation of the antitumor response against RNEU ( +) tumors, while the antitumor response against RNEU (-) tumors was not obvious, showing that the vaccine exhibited strong specificity [92]. Coumes et al. developed a peptide/polymer conjugate LD-indolicidin-PEG-PLA as an adjuvant in tumor immunotherapy, and found that it could effectively help induce helper T cell (Th1)-dominant antigen-specific immune response and anti-tumor ability [93]. Resiquimod (R848) is a toll-like receptor (TLR) 7/8 agonist and is highly efficient in cancer immunotherapy; however, its development into a drug is limited by tolerance induction and adverse reactions. Thauvin et al. treated cancer with modified PLA NPs supported with R848, and the results showed that NP had no toxicity to immune cells and could deliver immunoactive drugs more effectively [94]. Although PLA has not been sufficiently studied as well as other polymers, such as PLGA, for tumor immunotherapy, the accumulation of more scientific data and mechanism studies may likely improve its application in drug encapsulation and delivery; therefore, its future potential may be boundless.
2. *PLGA:* PLGA is an effective biodegradable polymer that allows controlled release of drugs. It is widely used to package tumor antigens and other immune adjuvants. Furthermore, it has good biocompatibility and can degrade and metabolize in vivo; additionally, the slow release rate can be up to several months [183]. The FDA and the European Medicines Agency have approved the application of PLGA through parenteral and mucosal pathways [184]. PLGA NPs are the most attractive polymer NPs in tumor immunotherapy. Chen et al. prepared the PLGA-ICG-R837 NP composed of PLGA-encapsulated ICG (indocyanine green, photothermal treatment material) and R837 (imiquimote, immune adjuvant) for the elimination of proto-cancer tumors. Adjuvant immunotherapy inhibits tumor spread and produces immune memory effects to inhibit tumor recurrence. The composite was triggered by a near infrared light and first performed photothermal treatment on the tumor site. The stimulated release of tumor-associated antigen interacted with R837 to produce a solid immune response and promote anti-CTLA-4 checkpoint inhibitor immunotherapies [95]. According to the study by Kim et al., incorporating TLR 7/8 agonist into PLGA NPs could enhance the expression of co-stimulatory molecules as well as antigen presentation in DCs compared to free agonists. Studies have shown that these NPs migrate to lymph nodes, trigger DC activation and amplification, and lead to improved CTL response, thereby improving the preventive and therapeutic efficacy of tumor models of melanoma, bladder cancer, and renal cell carcinoma [185]. Zhu et al. developed a pH-responsive NP mainly based on PLGA and CaCO_3_, which could achieve the effective co-encapsulation of the inhibition of adriamycin [an inducer of immunogenic cell death (ICD)] and alkylated NLG919 (aNLG919) (an indoleamine 2, 3-dioxygenase 1 inhibitor). The formulation of PLGA-CaCO_3_ NPs loaded with DOX/aNLG919 (DNCaNPs) caused ICD in cancer cells and decreased the production of an immunosuppressant molecule, kynurenine, through the inhibition of indoleamine 2, 3-dioxygenase 1. DNCaNPs presented effective accumulation in the tumor site and facilitated the loaded drug to spread within the tumor, as well as neutralize the acidic TME. Moreover, they relied on the combination of chemotherapy and immunotherapy to exert effective suppression of the growth of subcutaneous CT26 tumor models and in situ 4T1 tumor models (Fig. 8A, B) [96]. Koerner et al. developed a PLGA granule-based vaccine that was co-encapsulated in the PLGA granule with OVA and the double-stranded RNA adjuvant Riboxxim. PLGA granules triggered the highest adjuvant effect of Riboxxim, led to effective activation of mouse and human DCs, and elevated tumor-specific CD8 T cell response. Such PLGA pellet vaccines can delay tumor progression, restrain tumor metastasis, and prolong survival time of immunized mice. Their beneficial therapeutic efficacy is further enhanced by immune checkpoint inhibition, leading to the recovery of cytotoxic T lymphocyte response, and tumor ablation [97]. Podojil et al. modified the regimen of a PLGA NP (ONP-302) to achieve tumor control. Infusion of ONP-302 reprogrammed myeloid cells by activating STING/IL-15/NK cell mechanisms. Furthermore, it strengthened PD-1/PD-L1 expression in the TME, hence anti-PD-1 treatment was allowed in the B16.F10 melanoma model, which is usually not effective against PD-1 monotherapy [98]. Compared with other biomaterials, PLGA-based NPs have advantages of biocompatibility, biodegradability, and modification feasibility, which makes PLGA suitable for nanomedical development [186]. Nevertheless, high production costs, difficulty in scaling up, rapid in vivo degradation of non-coated NPs, and lower drug delivery efficiency still limit the PLGA delivery systems. Therefore, further research is needed in the future.
3. *PEG:* PEG is a representative polymer carrier for preparing NPs. It has low toxicity, no antigenicity, good amphiphilicity, and good biocompatibility, and has been approved by the FDA [187]. Notably, bioactive functional groups on PEG polymers can be modified to enhance the biological effect of NPs [188]. Recently, PEG-modified NPs have been used to enhance tumor immunotherapy. For example, Dane et al. combined STING-activated circular dinucleotides (CDNs) with PEG-enabled lipids and incorporated them into lipid nanodisks (LNDs). LNDs transporting CDN-PEG lipids intravenously demonstrated more effective tumor penetration compared to the most advanced liposomes, which could expose most tumor cells to STING agonists. LNDs appear to be a promising vector for the stable delivery of compounds to solid tumors, which can be used to enhance immunotherapy [99]. Chen et al. found that salvianolic acid B-loaded PEGylated liposomes (PEG-SAB-Lip) inhibited TGF-β1 secretion to affect the activation of tumor-associated fibroblasts, thereby leading to less deposition of collagen and more penetration of NPs in tumors. In addition, there were low Th2 and CXCL13 expressions and decreased macrophages of MDSC, Tregs, and M2 in the tumor area. The combination of PEG-SAB-Lip and docetaxel-loaded PEG-modified liposome markedly inhibited tumor growth. In addition, PEG-SAB-Lip could further prevent a tumor from metastasizing to the lung [100]. Liang et al. developed a cascade of pH-activated supramolecular nanoprodrugs (PDNP) with step-down size properties as a pyrolytic inducer that promotes anti-tumor immune responses. PDNPs include multiple PEG and DOX drug-polymer mixed repeat blocks.

**Fig. 9:**
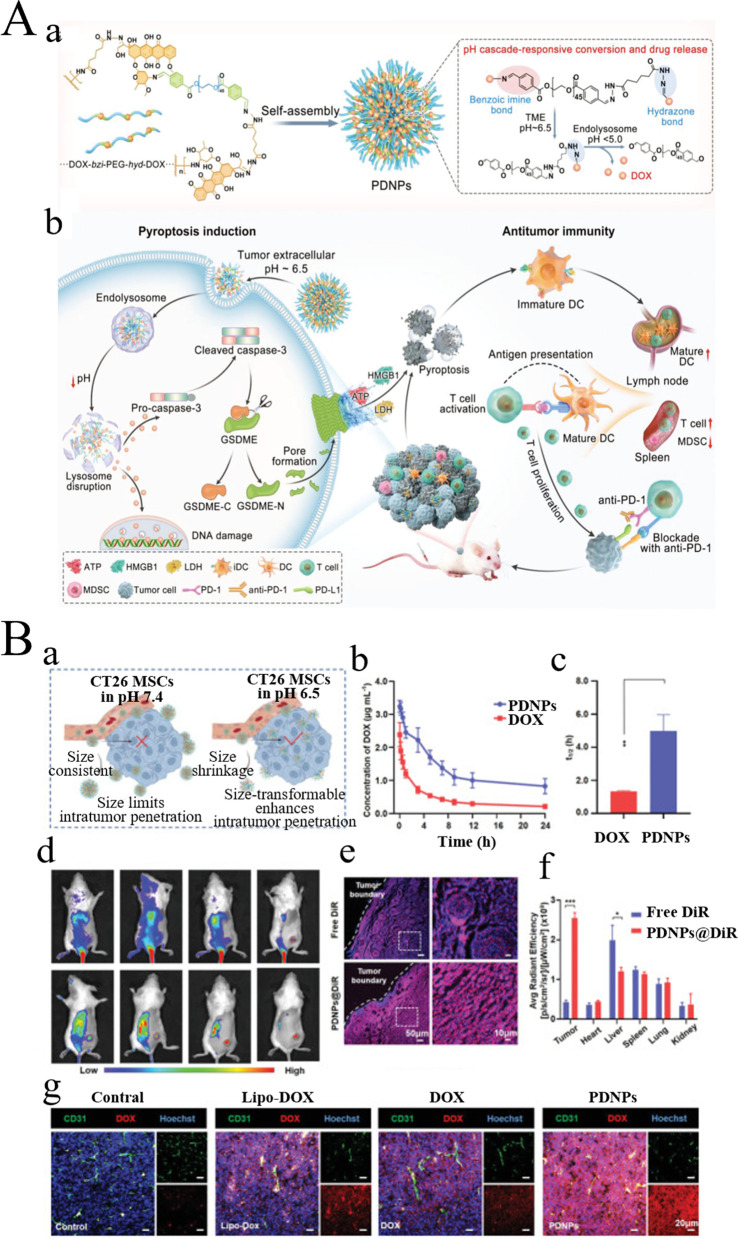
Schematic diagram of PEG based biomaterials for tumor immunotherapy. **A** Schematic illustration of size‐transformable supramolecular nanoprodrug (PDNP). (a) Structure of PDNPs and the mechanism of pH cascade‐responsive conversion and drug release. (b) Schematic diagram of anti-tumor effect of PDNP. **B** Antitumor efficacy of PDNPs. (a) Schematic illustration of PDNPs for enhanced intratumor penetration in CT26 MCSs. (b) Plasma concentration of DOX versus time of PDNPs upon administration of PDNPs and free DOX in S.D. rats. (c) The circulating half‐life (t 1/2) of PDNPs and free DOX. (d) In vivo NIR imaging of free DiR and PDNPs@DiR in CT26‐bearing mice. (e) CLSM images of the distribution and penetration of PDNPs in the slices of CT26 tumor tissue. (f) Quantitative analysis of the excised tumors and organs at 48 h postinjection. (g) The immunofluorescence staining of excised tumor tissues after treatments. (Reproduced with permission from [101] Copyright © 2022, Advanced Science)

**Fig. 10:**
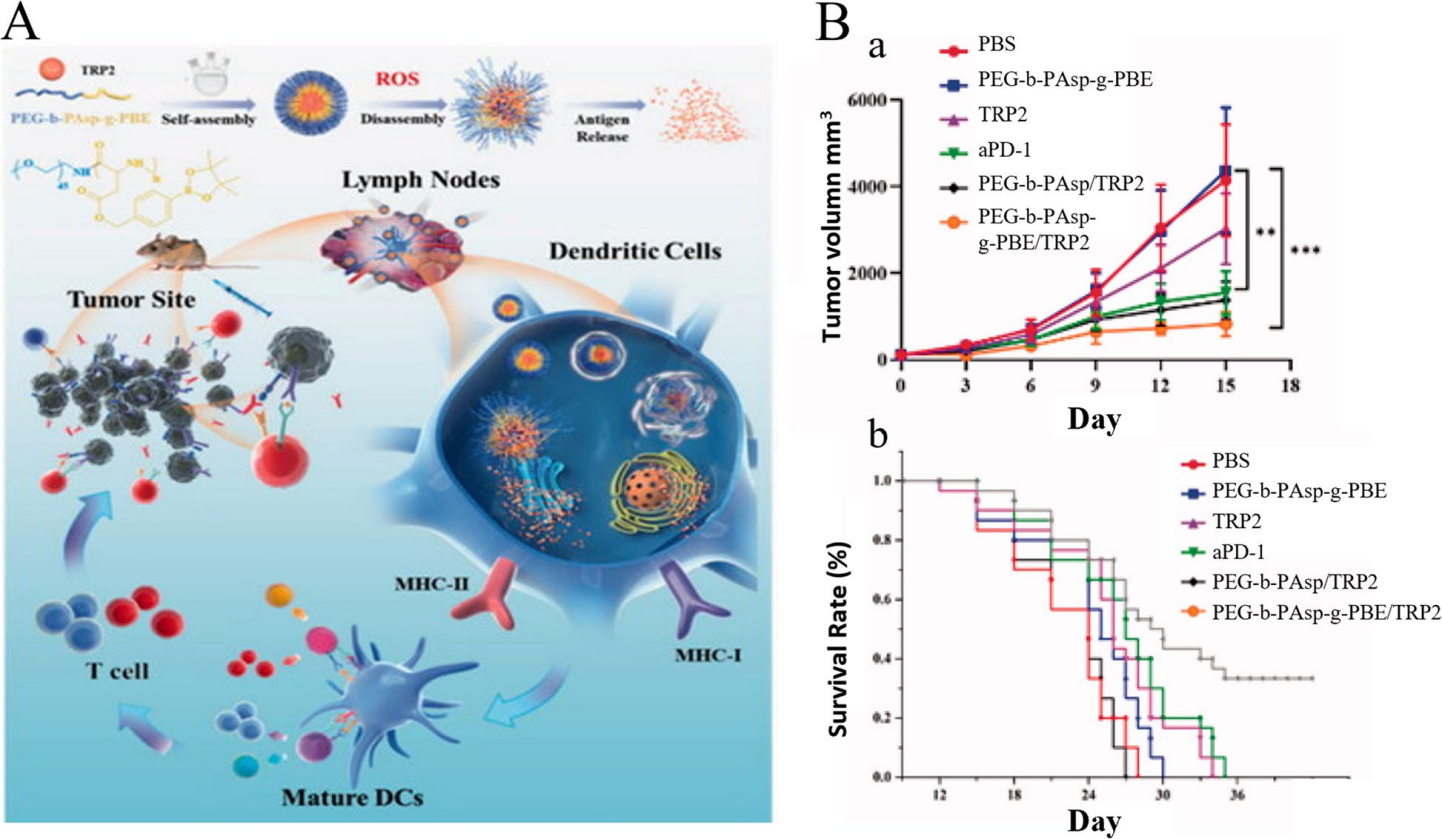
Schematic diagram of PEG-b-PAsp-g-PBE/TRP2 for tumor immunotherapy. **A** Schematic diagram of the nanovaccine delivery system PEG-b-PAsp-g-PBE/TRP2. **B** Antitumor effect of PEG-b-PAsp-g-PBE/TRP2 vaccine against B16F10 melanoma cells. (a) average tumor growth curves. (b) Survival data of mice. (c, d) The representative flow cytometry images and statistic data of (c) CD8.^+^ T cells and (d) Treg cells in the spleen at the end of the study. (Reproduced with permission from [102] Copyright © 2022, Drug Delivery)
  Qian et al. prepared microenvironment-responsive deformable (DMP@NPs) self-assembled from a tumor acid reaction polypeptide checkpoint inhibitor polymer (PEG-DMA-DPA-1) and ICD-strengthened drug combination. The contain a specific amount of mitoxantrone and proanthocyanidins, and c improve immunotherapy by synergically regulating the TME. After PEG-DMA-DPA-1 cleavage was triggered by tumor acidity, DMP@NPs underwent a special “dome-ring deformation” dissociation to gradually release the peptide checkpoint inhibitors DPA-1, mitoxantrone, and proanthocyanidins. The intravenous injection of DMP@NPs caused the reprogramming of the local TME in CT26 tumor-bearing mice and more visible DC activation and T cell infiltration. Synergistic immunonanomaterials DMP@NPs are capable of improving the efficacy of colorectal cancer immunotherapy and weakening its toxicity and side effects [103]. Zong et al. designed a ROS-responsive self-amplified chain-breaking poly (thioacetal) [represented by TA-CA], which conjugated DOX and amino terminated methoxylpolyethylene (mPEG-NH2) on the side chain of TA-CA to obtain an amphiphilic polymer (represented by TA-CA-DOX-PEG). NPs called TCD could be self-assembled in water-based media and accumulate in tumor tissues. After ingestion by cancer cells, endogenous ROS triggerred TA lysis and CA release, thereby producing a larger number of ROS through mitochondrial dysfunction, leading to exponential polymer degradation cascade and the rapid release of DOX in a self-amplifying manner. Furthermore, amplified oxidative stress may synergistically promote DOX-induced ICD to contribute to a more effective chemical immunotherapy [104]. The application of PEG in drug delivery systems is at the forefront of global pharmaceutical development [189], and more powerful and safer PEG-based products can be applied in tumor immunotherapy in the future.
4. *PEI:* PEI serves as a type of a cationic polymer that has recently been extensively highlighted in drug loading. It has become a research hotspot because of its high drug loading efficiency, easy modification of particles, and good biocompatibility [190]. Recently, Yang et al. constructed PEI lipid NPs (PEI-LNP)/siRNA complex (EPV-PEI-LNP-siRNA) with PD-L1-siRNA therapeutic function and EGFR short peptide/PD-L1 double immune-enhancing function for preventing and treating EGFR-positive lung cancer. Accordingly, such NPs have the capability of targeting cells, causing PD-L1 downregulation in vitro, allowing immunostimulatory cytokines to be specifically expressed, serving as an efficient and safe nanovaccine for targeted therapy [105]. Cheng et al. used a polymer complex of PEI and oleic acid (PEI-OA) to develop PD-L1 and CD44-responsive multifunctional NPs and loaded it with two chemotherapy drugs (paclitaxel and chloroquine), one antigen (OVA), one immune enhancement agent (CpG), and one ICI (anti-PD-L1 antibody). As observed, PEI-OA could remarkably strengthen the drug loading capacity and encapsulation efficiency exhibited by the nanoplatform, and the anti-PD-L1 antibody led to an apparent increase in the cell intake compared to other therapeutic preparations. Moreover, the anti-PD-L1 antibody exerted an obvious inhibitory effect on primary BC and a positive impact on CD4^+^ and CD8^+^ T cell levels at the tumor site, while strengthening the tumor immune efficacy [106]. Xiao et al. reported that a PEI-based terpolymer hydrogel, polyvinylalcohol, and magnesium ion stimulator of adaptive immune response showed an intrinsic immunomodulatory function, which could reverse the immune "cold" phenotype to "hot" phenotype of mouse mammary tumors by upregulating PD-L1 expression and strengthening M1-like macrophage polarization. PEI hydrogel encapsulation of immune checkpoint blocking inhibitors-anti-PD-L1 antibody (α-PDL1) synergistically assisted in eliminating primary tumors and distant metastases and preventing tumor recurrence because of surgical resection (Fig. 11A, B) [107]. Li et al. loaded indoleamine 2, 3-dioxygenase inhibitors and TLR agonists into PEI-coated polydopamine NPs. The conveyance system was highly uniform, stable, biocompatible, and low in toxicity. In vitro studies showed that the system exerted an effective inhibitory impact on tumor proliferation and an inductive impact on BC cell apoptosis. In vivo studies showed that the system restrained BC growth, enhanced APC maturation and T lymphocyte differentiation, while inducing the body’s immune response [191]. Zhao et al. designed a binary nanovaccine BiVax that could rapidly prepare personalized vaccines by combining PEI-4BIMI (an azole molecular terminal cap PEI) with autologous tumor cell membrane protein antigen; the postoperative cure rate could reach 60% when used in combination with ICIs [192]. There are still some challenges in the application of PEI in tumor immunotherapy. For example, the operation is difficult, the preparation is complex, and the cytotoxicity is confirmed [193]. Therefore, further exploration is needed in the future.

**Fig. 11:**
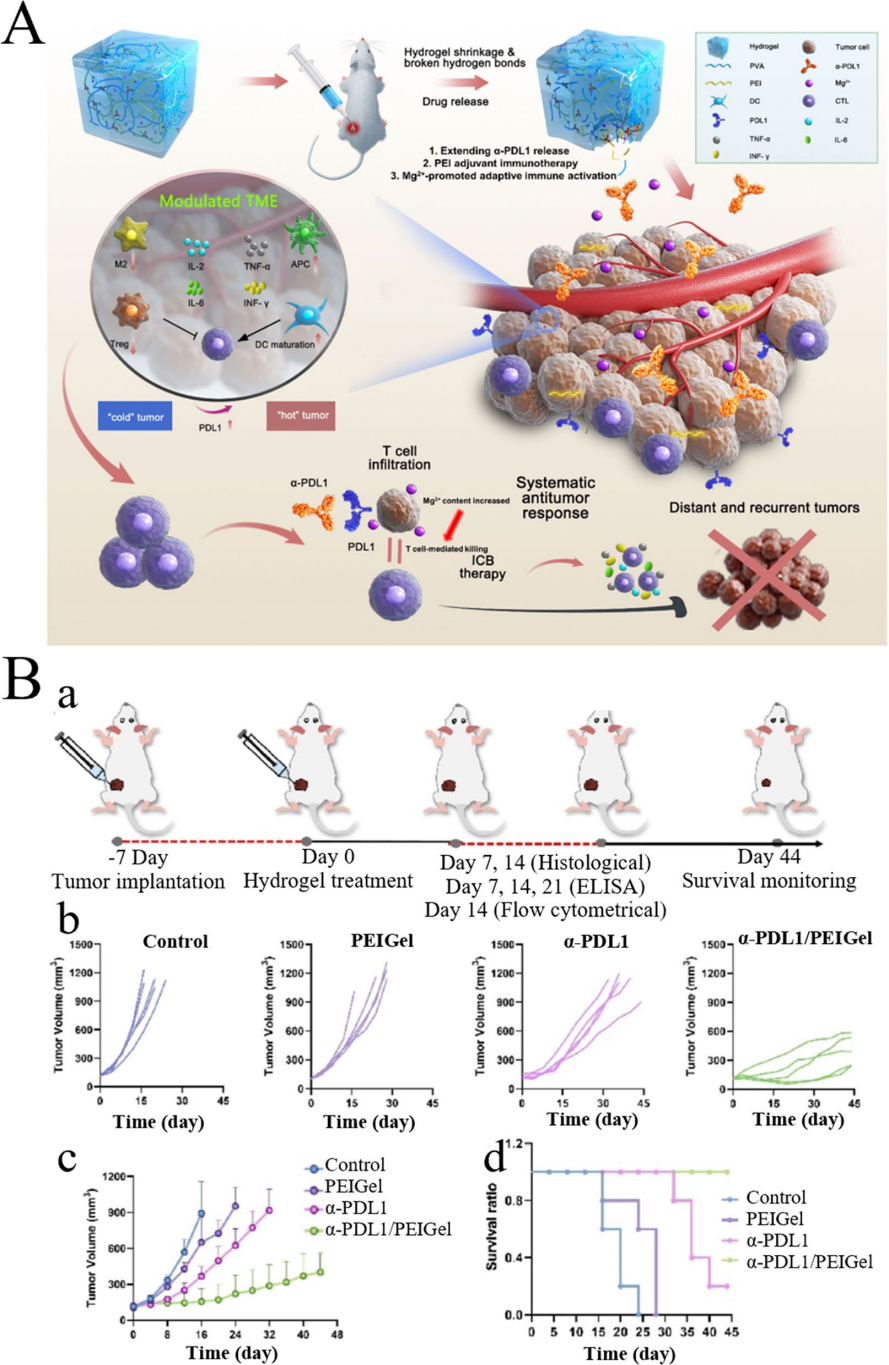
Schematic diagram of PEI based biomaterials for tumor immunotherapy. **A** Schematic illustration of α-PDL1/PEIGel for boosting and enhancing ICB therapy. **B** Antitumor efficacy of the α-PDL1/PEIGel in a 4T1 allograft murine model. (a) Schematic diagram of the animal experiment. (b) Individual tumor volumes. (c) Tumor growth curves of 4T1 tumor-bearing mice with different treatments for 44 days. (d) Kaplan—Meier survival analysis of 4T1 tumor-bearing mice. (Reproduced with permission from [107] Copyright © 2022, Elsevier)

Polymer NPs are adjuvants and delivery systems specific to molecules that stimulate immune response, which have enjoyed extensive application in cancer immunotherapy. Some functional polymer NPs are capable of activating immune response and enhancing the responses to relevant tumor cells. As an efficient delivery system that protects the contents from environmental impact and provides a sustained and adjustable release rate through targeting strategies, polymer NPs are a strategic area for future development in tumor immunotherapy [194].

##### Natural biomacromolecular materials

High polymers such as PLA and PLGA have good biocompatibility; however, they produce acid degradation products to affect local pH, resulting in the degeneration of encapsulated drugs and reduced drug efficiency [195]. In contrast, all natural polymer materials have good biocompatibility and biodegradability; as a result, they are suitable for selection as nanomaterials. Natural biomolecular materials can effectively improve drug utilization, reduce toxic side effects, achieve targeted drug transportation, and reduce pain for patients [196, 197]. According to the source of different materials, natural polymer materials include polysaccharide, silk fibroin protein, and gelatin.

**Fig. 12:**
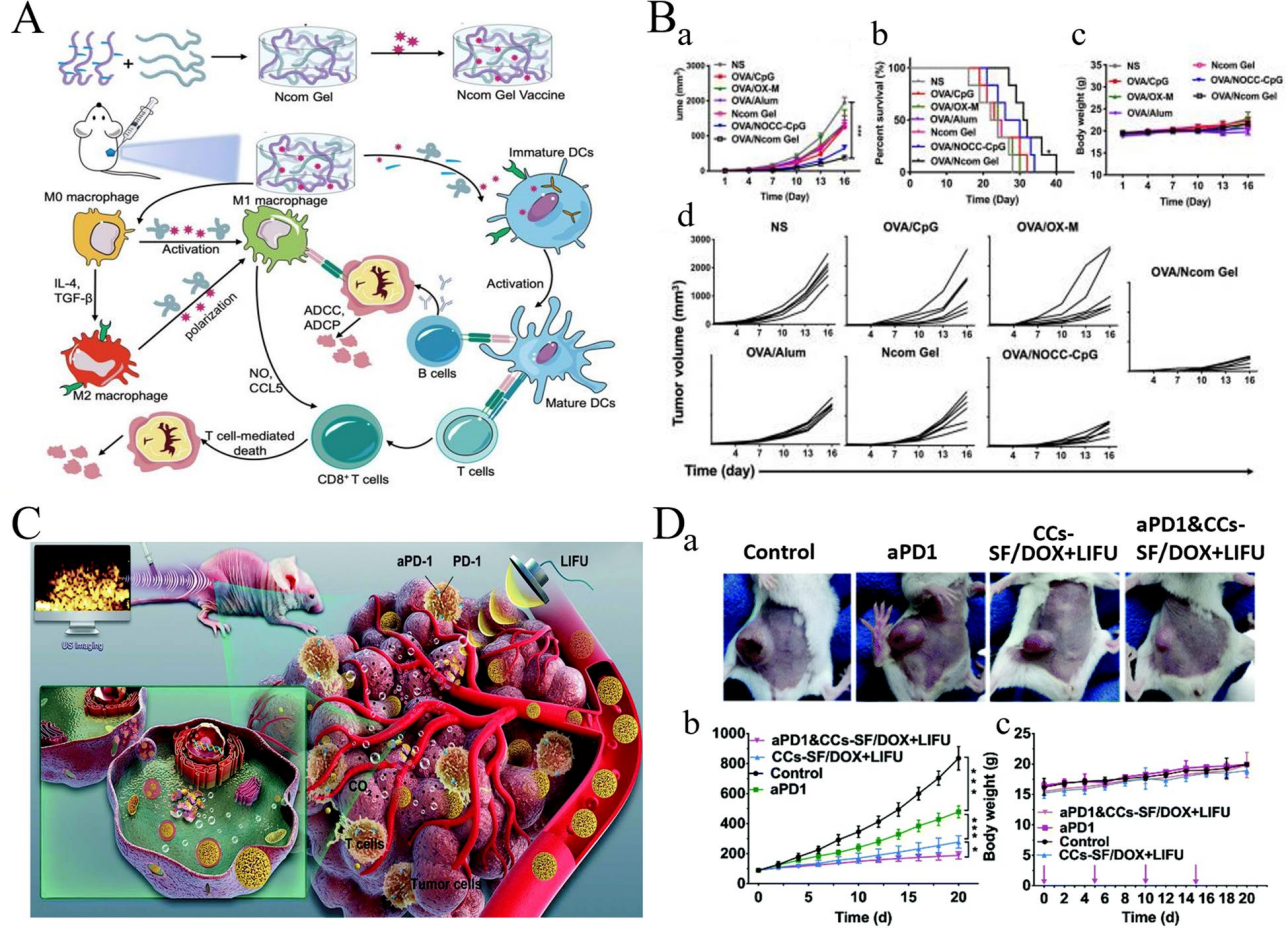
Schematic diagram of polysaccharide or silk fibroin based biomaterials for tumor immunotherapy. **A** Schematic diagram of preparation and action steps of NOCC-CpG/OX-M hydrogel (Ncom Gel) vaccine. **B** Antitumor efficacy of the Ncom Gel vaccine in C57BL/6 melanoma mice. (a) Average tumor growth curves of B16F10-OVA tumor-bearing mice after treatment. (b) Survival curves of mice after treatments. (c) Body weight curves during the treatment. (d) Individual mouse tumor growth curves with different treatments. (Reproduced with permission from [111] Copyright © 2021, Theranostics) **C**. Schematic illustration of the local chemotherapy-therapeutic EVs-synergistic immunotherapy. **D** Synergistic efficacy of aPD1 & CCs-SF/DOX NPs to trigger antitumor immune response. (a) Representative pictures of tumors on Balb/c mice at day 20. (b) Tumor growth curves and (c) body weight changes of Balb/c mice. (Reproduced with permission from [113]  Copyright © 2020, Royal Society of Chemistry) [113]

**Fig. 13:**
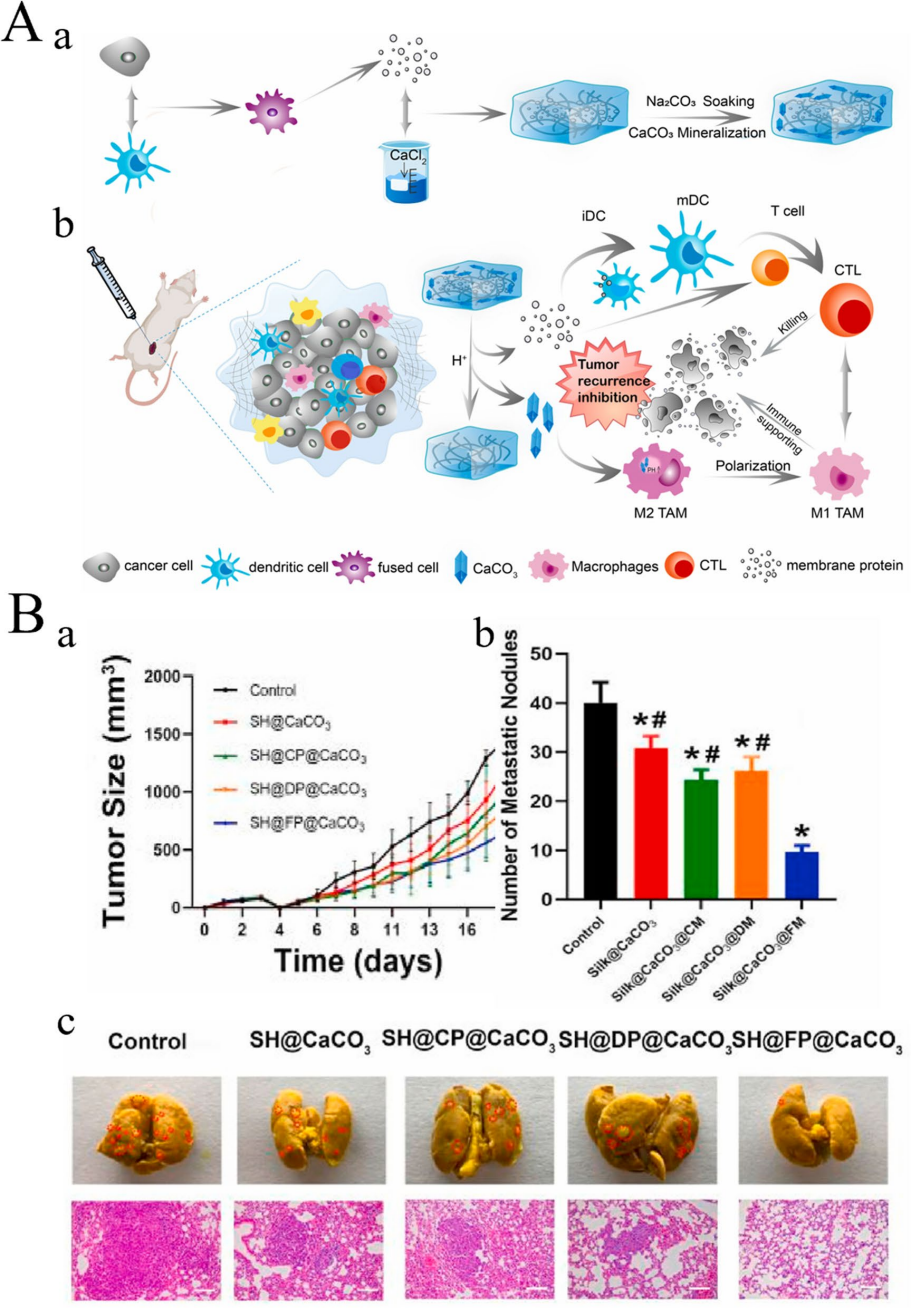
SH@FP@CaCO3 vaccine for tumor immunotherapy. **A** Illustration of the SH@FP@CaCO3 vaccine that inhibits tumor recurrence and metastasis after surgery. **B** Immunotherapeutic gel for inhibiting tumor recurrence. (a) The tumor sizes in different treatment groups. (b) The number of lung metastasis nodules after different treatments. (c) Representative photos and H&E staining images of the lung isolated from the mice of different groups. (Reproduced with permission from [114] Copyright © 2022, Elsevier)

**Fig. 14:**
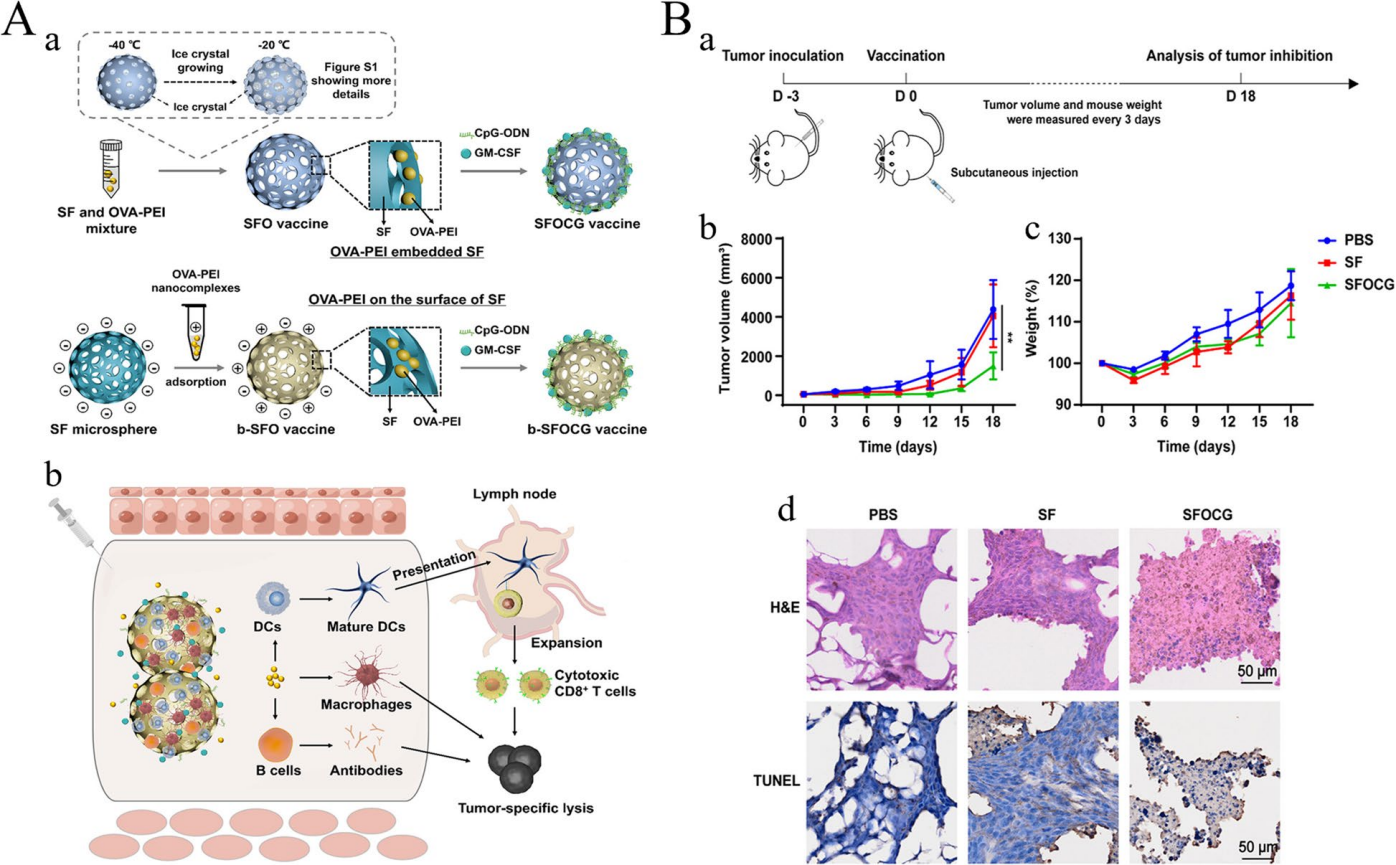
Macroporous SF microsphere-based vaccines for tumor immunotherapy. **A** Schematic diagram of injectable macroporous SF microsphere-based vaccines. (a) Preparation of macroporous SF microsphere-based vaccines. (b) Mechanism of the vaccine in triggering tumor-specific immune responses to kill tumors. **B** The antitumor capacity of SFOCG vaccine. (a) The scheme of SFOCG inhibiting B16F10-OVA tumor. (b) Tumor growth and (c) body weight of the mice bearing B16F10-OVA tumors. (d) H&E staining and TUNEL staining of B16F10-OVA tumors in the mice at day 18. (Reproduced with permission from [115] Copyright © 2022, American Chemical Society)

**Fig. 15:**
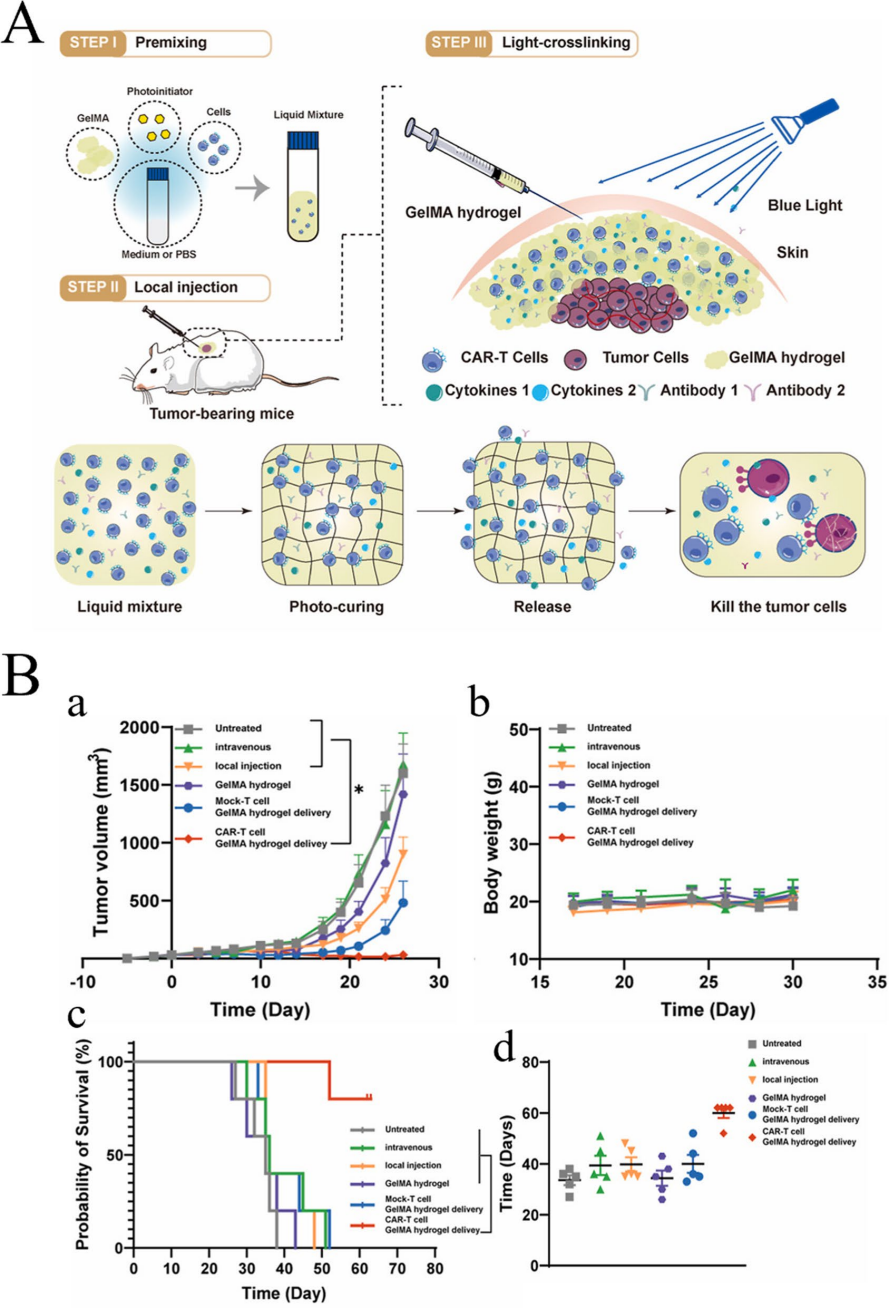
Surgery-free injectable CAR-T cells delivery system for tumor immunotherapy. **A** Schematic diagram of the installation process of the injectable delivery system. **B** Anti-tumor efficacy of the delivery system in vivo. (a) The average tumor growth curves of various treatment groups. (b) The average body weight of mice post different treatments. (c) The survival curve of mice post different treatments. (d) The average survival time of various treatment groups. (Reproduced with permission from [208] Copyright © 2022, Elsevier)

**Fig. 16:**
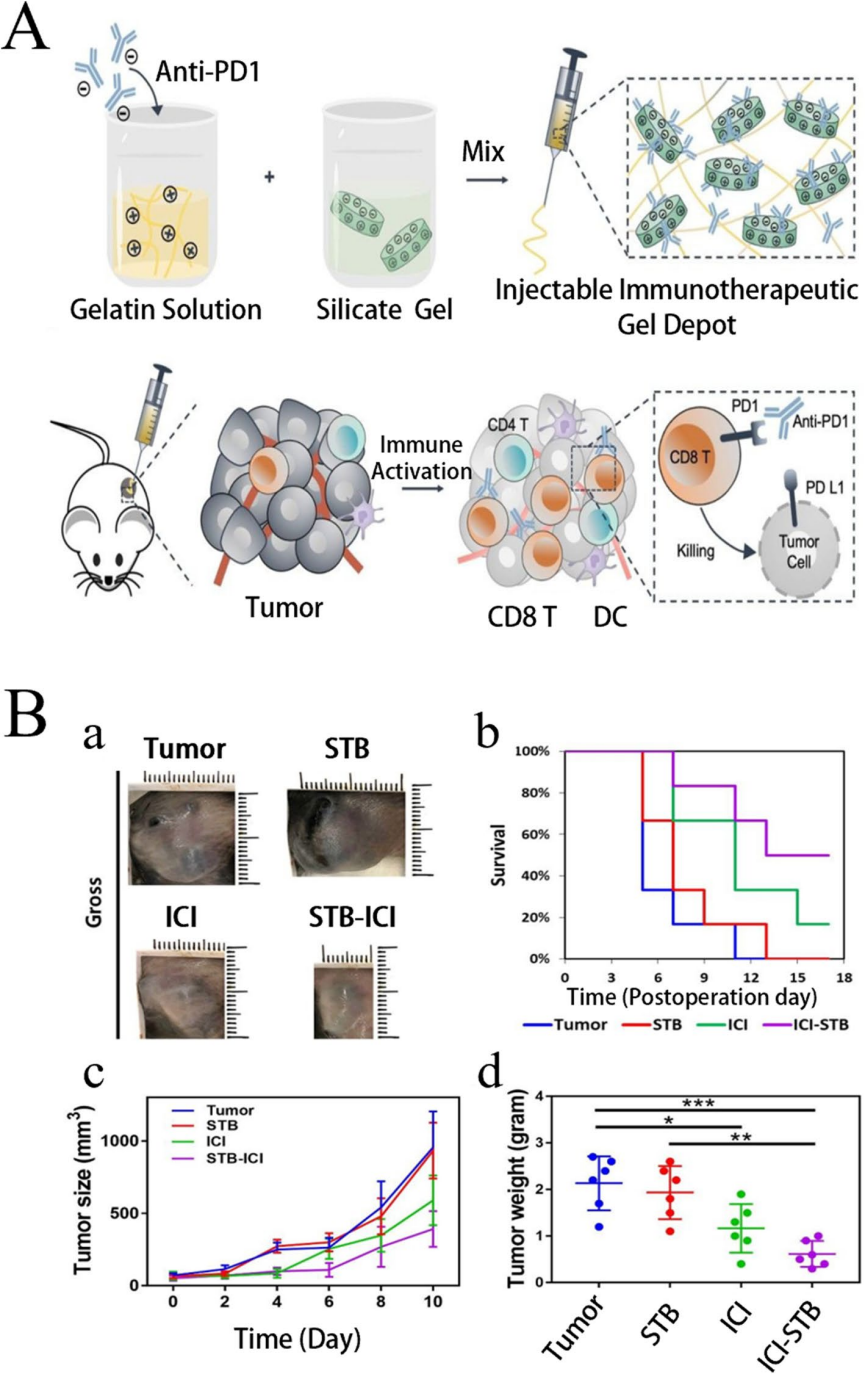
STB-mediated ICB for tumor immunotherapy. **A** Schematic illustration of STB-mediated ICB. **B** Antitumor efficacy of STB-ICI. (a) Gross appearances of the tumors. (b) Survival rate analysis. (c) Tumor growth curve. (d) Tumor weight at the day of sacrifice (day 10 post operation) (Reproduced with permission from [118] Copyright © 2022, American Chemical Society)

1. *Polysaccharides:* Natural polysaccharides are biosynthetic polymer compounds composed of multiple monosaccharides covalently linked by glycosidic bonds. They possess various biological properties, particularly anti-tumor effects [198]. Compared with existing tumor therapy drugs, natural polysaccharides have become a research hotspot in the field of anti-tumor therapy owing to their safety and high efficiency. Nehal et al. investigated the novel polysaccharide sodium alginate/κ-carrageenan microcapsules, which served as an oral targeted drug delivery system in immunotherapy for colon cancer. There was a considerable increase in the number of CD16CD56 NK cells, and the activated NK cells exerted 74.09% cytotoxic effect on Caco-2 cells. Most of these cancer cell populations remained in the G0/G1 phase [108]. Hyaluronic acid (HA) acts as a type of acidic mucopolysaccharide. It is water-soluble, biocompatible, and biodegradable, and has CD44-targeting properties [199]. Kim et al. developed a polymer nanoconjugated compound (PEG-HA-OVA/PPLs), composed of a siPD-L1-based polymerase, PEG-alized HA as a CD44-targeting component, and OVA as a model foreign antigen. PEG-HA-OVA/PPLs migrated to CD44 tumor cells and CD44 DCs, resulting in high cross-expression of OVA and downregulation of PD-L1 in both cell types. This conjugate brought about a strong rejection by OVA-specific T cells of the disinfected tumor cells, while reprogramming the TME for stimulating an intense T cell response against endogenous tumor antigens, followed by the generation of durable protective immunity [109]. Natural polymeric polysaccharide hydrogels are biodegradable and biocompatible, and have wide sources and low cost, contributing to their special advantages in drug delivery [200]. Li et al. focused on the development and optimization of a PD-1/PD-L1 blocking peptide, OPBP-1, which was then loaded onto a hydrogel oral delivery system based on TMC (N, N, n-trimethyl chitosan). To maximize the oral bioavailability exhibited by peptide drugs and significantly inhibit tumor growth [110], a spontaneous multifunctional hydrogel (Ncom Gel) vaccine was developed. The formation was based on the Schiff base reaction between the CpG-modified carboxymethyl chitosan (NOCC-CpG) and the partially oxidized mannogan (OX-M), (NOCC-CpG/OX-M), which is capable of amplifying innate immune responses and utilizing innate immunity for the initiation and maintenance of intense adaptive immune responses (Fig. 12A, B) [111].
2. *Silk fibroin (SF):* SF, a natural polymer, is water-soluble, biodegradable, biocompatible, and structurally modified, and can serve as a carrier material for drug delivery [201]. The encapsulation of NPs formed by these drugs using SF as the carrier has a certain passive targeting through enhanced penetration and retention effect [202] and active targeting if SF is structurally modified, thus reducing systemic toxicity. Ornell et al. used freeze-dried SF foam-based local delivery of dinutuximab to treat a mouse model of in situ neuroblastoma and achieved a significant reduction in tumor growth [112]. Tan et al. constructed an SF-based DOX pre-loaded calcium carbonate incorporating tumor-derived extracellular vesicles for the triple therapy of “local chemotherapy-EVs treaty-Synergistic immunotherapy”. This system could promote M1-like macrophage polarization and reverse immunosuppressive TME, in addition to PD1/PD-L1 checkpoint blocking combined with immunotherapy (Fig. 12C, D) [113]. Song et al. developed a percutaneous tumor vaccine delivery system with SF and polyvinyl alcohol composite nanofiber patch loaded with mannose-based PEI-modified ethanoles called Eth PEIman. The system could target DCs percutaneously and remarkably restrain tumor growth. In addition, combining the vaccine loaded patch with PD-1 brought about a synergistic effect on melanoma resistance [203]. Huo et al. synthesized the CaCO_3_ biomineralized hydrogel DC vaccine (SH@FP@CaCO_3_) by immobilizing the membrane protein of 4T1 cell-DC fusion cells into a biomineralized SF hydrogel. The SF hydrogel vaccine showed excellent immunoactivation effect by simultaneously enhancing immunogenicity and reversing immunosuppressive TME, which provides a promising strategy for cancer immunotherapy (Fig. 13A, B) [114]. Fang et al. developed an injectable macroporous SF microsphere loaded with antigen and immune adjuvant, which could inhibit established tumors with only one injection. It had a macroporous structure, which assisted in recruiting immune cells and activating DCs, hence a proper immune microenvironment was formed and a strong humoral and cellular immunity was further induced. The researchers also enhanced the vaccine by simply attaching the antigen to the SF microspheres. This effectively inhibited tumor growth by increasing cytotoxic T lymphocyte response (Fig. 14A, B) [115]. SF-based tumor immunotherapy has broad application prospects, but the compatibility and safety of SF-loaded NPs have not been comprehensively discussed [202, 204]. Hence, large-scale baseline and practical research is warranted.
3. *Gelatin:* Gelatin is water-soluble, acquired from collagen hydrolysis by acids, bases, or enzymes, and mainly found in the skin, bone, and connective tissue of animals (fish and insects) [205]. As a biomedical material, gelatin possesses biocompatibility, low immunogenicity, and ease of operation, which allow for its broad application in biomedicine [206]. In 2017, Oh et al. used gelatin-based hydrogel-mediated co-delivery of oncolytic AD and DC to deliver IL-12 and GM-CSF for immunotherapy, effectively retaining the two therapies in tumor tissues as well as inducing a useful anti-tumor immune response for a longer period through a single administration [207]. Using a similar principle, Du et al. recently encapsulated gelatin-based hydrogels that can co-deliver oncolytic adenoviruses equipped with IL12, IL15, and CIK cells to enhance and prolong their antitumor effects after a single intra-tumor injection [116]. Suraiya et al. investigated an injectable, gelatin-based microhydrogel system that encapsulated and delivered effective CAR-T therapy. Microgel-recovered CAR-T cells could completely eliminate tumor cells, showing a cytotoxic effect targeting human ovarian cancer in vitro and on 3D tumor spheres [117]. Subsequently, Zhou et al. similarly used injectable and photocurable geline-methacrylate hydrogels as CAR-T cell repositories to form injectable CAR-T geline-methacrylate hydrogel delivery (i-GMD) systems. The results showed that CAR-T cells in the system could expand normally, release continuously, and play an antitumor role in vitro. Injecting i-GMD matrix around the tumor showed an enhanced antitumor effect and significantly extended survival in mice compared to local or intravenous CAR-T solution (Fig. 15A, B) [208]. Wu et al. locally delivered anti-PD-1 using a gelatin-based shear-thinned biomaterial (STB). In a mouse melanoma model, STB-ICI injection exerted a negative impact on tumor growth and a positive impact on CD8 T cell levels in peripheral blood. STB-ICI was also capable of elevating levels of tumor-infiltrated CD4 helper T cells and CD8 cytotoxic T cells, and inducing tumor death. The gelatin-based minimally invasive strategy simply and effectively assisted in the local delivery of ICIs (Fig. 16A, B) [118]. Relative to other biological materials, gelatin possesses a large surface area to volume ratio and strong porosity. The pore size is controllable, hence the drug solubility can be accelerated and drug delivery efficiency can be improved. However, research is ongoing to release more variety of biomolecules from gelatin carriers.

##### Cell-derived bioactive materials

Although nanocarriers have become a key part of tumor immunotherapy, their application in vivo is still faced with risks such as immune clearance [209]. To deal with these risks, a strategy known as “camouflage” has been introduced. Liangfang et al [210]. first proposed this strategy, which involved the transfer of the recognition and dialogue mechanism of red blood and immune cells into nanomaterials through cell membrane modification; this was done to obtain camouflage to avoid the clearance of nanomaterials by immune cells, and thus derive the bionic delivery platform of nanomaterials modified by the cell membrane. This platform not only contained the physical and chemical properties of nanocarriers but also biological properties similar to natural cells, making it suitable for tumor immunotherapy [211].

Cell membranes have different sources that provide different biological functions. The corresponding cell membrane-modified nanocarriers can be selected according to specific requirements to keep the original functions unchanged and simultaneously transfer the biological properties of cell membranes to achieve better tumor immunotherapy effects [212]. Based on the research on red cell membrane-modified nanocarriers, researchers have developed a series of nanocarriers based on platelet, macrophage, dry, tumor, and modified bacterial membrane. These novel biomimetic nanocarriers have more diversified functions that involve not only long blood circulation and drug loading functions but also the integration of active targeting of immune antigen, controllable slow release, and other characteristics that enhance their application potential in tumor diagnosis and treatment [213].

**Fig. 17:**
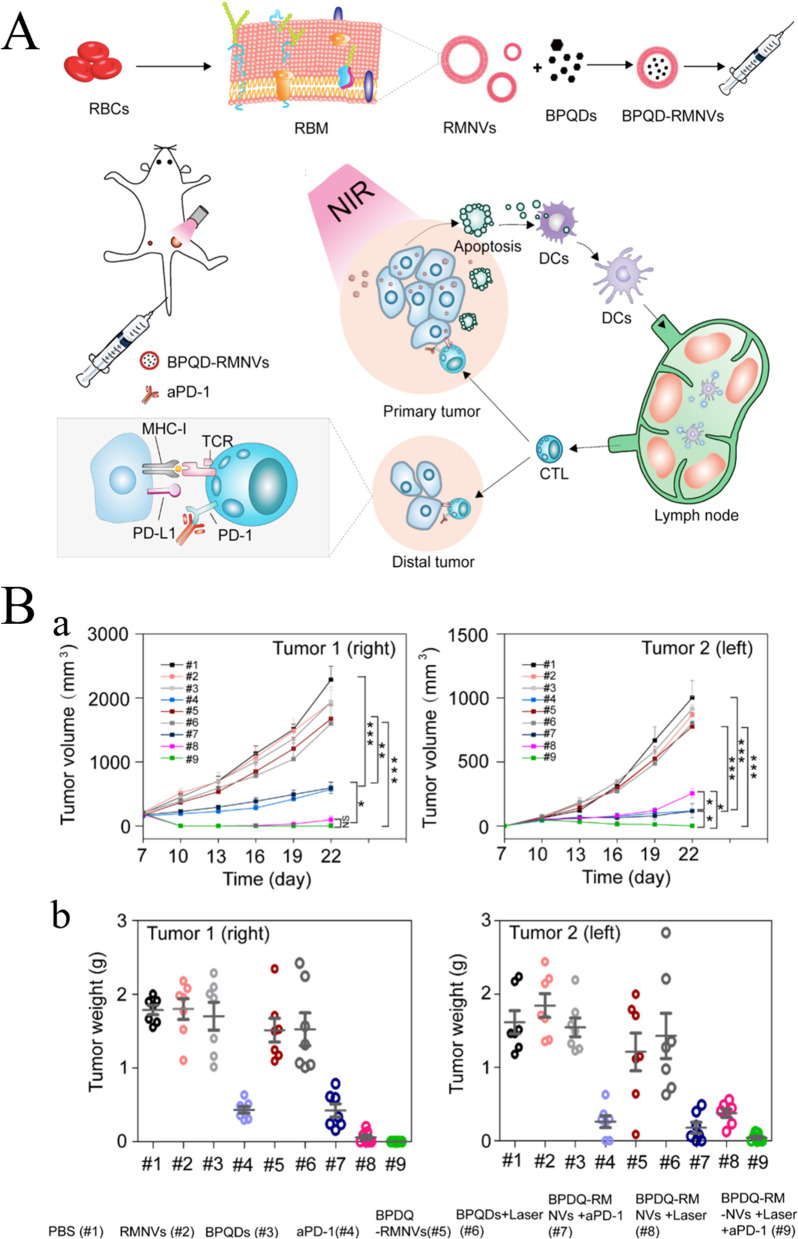
BPQD-RMNVs for tumor immunotherapy. **A** Schematic illustration of PTT and immunotherapy mediated by BPQD-RMNVs and aPD-1. **B** In vivo anti-tumor effect of BPQD-RMNV. (a) Average tumor volumes of the treated mice in the different groups. (b) Weights of tumors extracted from euthanized mice in the different groups. (Reproduced with permission from [119] Copyright © 2019, Elsevier)

**Fig. 18:**
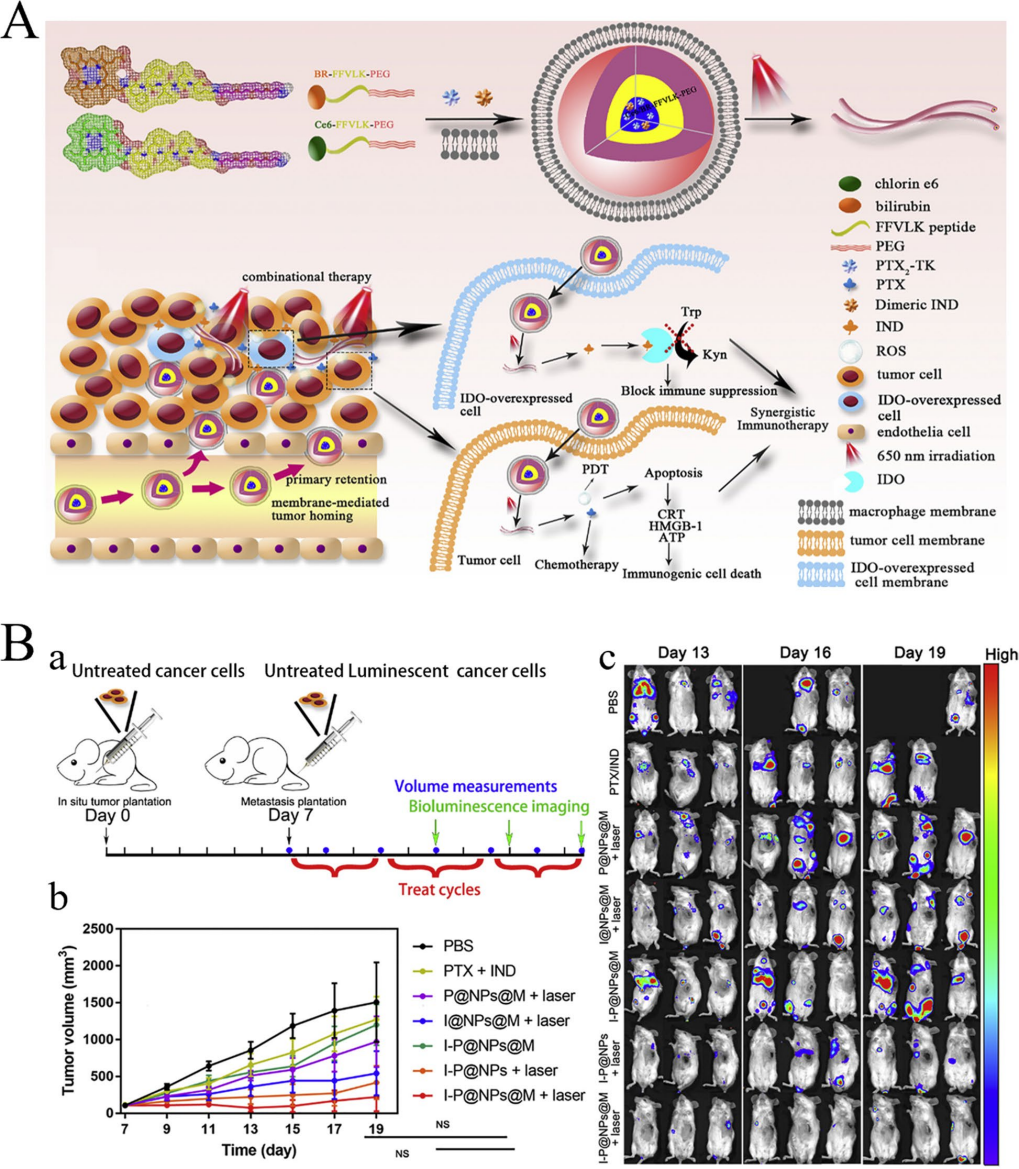
Schematic diagram of I-P@NPs@M for tumor immunotherapy. **A** Illustration of the construction and function steps in tumor tissue of I-P@NPs@M. **B** Progress steps and main results. (a) The red braces indicates the treat cycles. The blue dots indicate the volume record and the green arrows indicates the BLI. (b) The record of tumor volume reflects the suppression of in situ breast cancer by drugs. (c) Tumor imaging at different times in each treatment group. (Reproduced with permission from [123] Copyright © 2020, Elsevier)

**Fig. 19:**
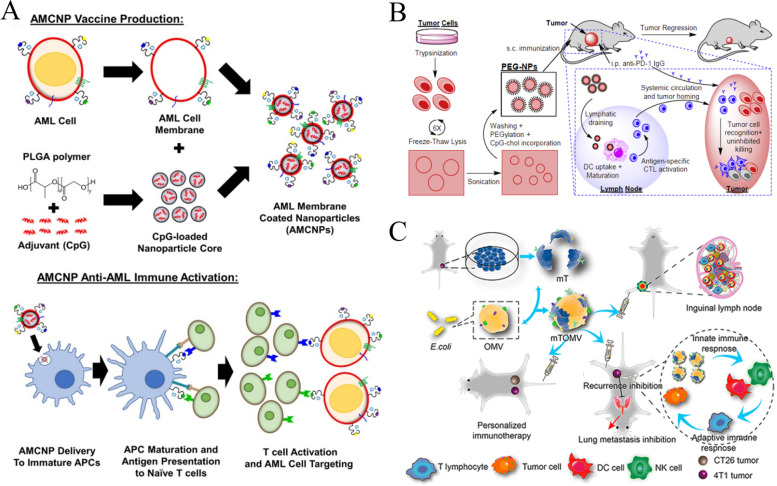
Schematic diagram of cell derived biomaterials for tumor immunotherapy. **A** Schematic of AMCNPs production and anti-leukemic vaccination. (Reproduced with permission from [127] Copyright © 2022, Springer Nature) **B**. Schematic diagram of PEG-NPs preparation and action. (Reproduced with permission from [128] Copyright © 2018, Elsevier) **C**. Schematic diagram of the action principle of mTOMV. (Reproduced with permission from [130] Copyright © 2021, American Chemical Society)

1. *Red blood cell (RBC) membrane:* RBCs are the most prevalent blood cells in humans and the main medium for blood transport of oxygen in vertebrates; furthermore, they have immune functions [214, 215]. The isolated RBC membrane can better protect the activity of the substance being transported, allowing it to have a longer and more controlled life cycle and avoid clearance by the immune system. RBC membrane-modifed nanocarriers are highly valuable transport carriers [216]. Liang et al. developed a bionic black phosphorus quantum dots (BPQDs) nanopreparation for treating TNBC, which used RBC membranes to cover BPQDs and form a BPQD-RM nanovesicle bionic preparation that showed longer cycle time and better tumor enrichment in vivo (Fig. 17A, B) [119]. Xiong et al. conducted the fusion of a murine-derived ID8 ovarian cancer cell membrane with an RBC membrane to generate a magnetic NP Fe3O4-ICG@IRM for application in the co-treatment of ovarian cancer. This NP demonstrated highly specific self-recognition of ID8 cells in vitro and in vivo, which strengthened antitumor immunotherapy specific to primary and metastatic tumors [120].
2. *Platelet membrane:* Platelets are responsible for hemostatic healing and immune response in the human body. As related antigens and proteins widely spread on platelets, they play an important role in anti-tumor metastasis [217]. The membrane surface modification of platelets has the same effect as that for RBCS, which is the lengthening of nanocarriers’ blood circulation time and evasion of immune clearance [218]. Recently, Bahmani et al. adopted platelet membrane coated with TLR agonists to construct the NP PNP-R848. Intratumor administration of PNP-R848 remarkably enhanced local immune activation and resulted in thorough tumor regression in colorectal tumor models. In addition, treatment of invasive BC models with intratumoral PNP-R848 delayed tumor growth and inhibited lung metastasis [121]. Further, Yan et al. constructed platelet membrane-coated NPs for the combined chemophotodynamic and immunotherapy of melanoma. Relying on the platelet membrane, NPs exhibited outstanding long cycle effect and strong tumor-targeting ability, capable of effectively solving the problem of low drug delivery efficiency [122].
3. *Macrophage cell membrane:* As the body’s first line of defense, macrophages are activated, which then recruit, engulf, and digest harmful invaders after detecting the signal of infection or tissue damage. By using specific recognition proteins on the macrophage cell membrane, nanocarriers can effectively improve their aggregation in specific parts of the body and enhance the efficiency of targeted drug delivery [219]. Liu et al. reported a laser-responsive and shape-changing nanomedicine I-P@NPs@M, which is coated with macrophage cell membrane and is capable of enhancing the long cycle and tumor-targeting ability of nanomedicine. Through the combined application of chemotherapy, photodynamic therapy, and immunotherapy, I-P@NPs@M has shown good anti-tumor effect and significant inhibitory effect on lung metastases (Fig. 18A, B) [123]. Chen et al. proposed a tumor-associated macrophage cell membrane (TAMM), which originates from primary tumors and has special antigen-homing affinity and immunocompatibility. TAMM coated with NPs can activate macrophages from M2-like phenotype to M1-like state and induce ICD; this promotes the production of tumor-specific effector T cells in metastatic tumors through activation of antigen-presenting cells, thus improving the efficiency of anti-tumor immunity [124].
4. *Stem cell membrane (SCM):* Stem cells are a class of cells that are not fully differentiated and have the potential to regenerate various tissues and organs [220]. In addition, bone marrow mesenchymal stem cells have low immunogenicity [221] as well as migrating and homing abilities [222]. NPs camouflaged with SCMs were found to migrate to tumor sites and effectively inhibit tumor growth [223]. By exploiting the tumor-targeting properties and biocompatibility of SCMs, Mu et al. constructed SCM-camouflaged polydopamine NPs carrying DOX and PD-L1 siRNA (PDA-DOX/siPD-L1@SCM) for the treatment of prostate cancer bone metastases. In vitro and in vivo experiments have shown that PDA-DOX/siPD-L1@SCM NPs can effectively enhance blood retention of the drug, improve tumor site accumulation, and exhibit good antitumor efficacy [125]. Recently, Xie et al. developed human umbilical cord mesenchymal SCMs coated with hollow manganese dioxide (HMnO_2_) and TAT peptide as a high-performance nuclear targeted drug delivery system (HMnO_2_-TAT@PTX NPs), which was used to treat NSCLC. The experimental results showed that the platform could effectively promote DC maturation and effector T cell infiltration, thereby effectively inhibiting tumor growth, recurrence, and metastasis [126]. The targeting and immunocamouflage properties of SCM surface proteins have greatly improved the targeting and biocompatibility of NPs, providing new targeting vectors for drugs and genes for tumor immunotherapy, which have good developmental prospects.
5. *Tumor cell membrane:* Tumor cells have a variety of unique functions, like strong replication potential, cell death resistance, immune escape ability, long cycle time, and homologous binding ability, which make them a new modification material for the design of anti-tumor therapeutic systems [224]. Tumor cell membrane modification may also be a novel personalized strategy for developing nanovaccines specific to different tumor types [225]. Johnson et al. developed and characterized an acute myelocytic leukemia (AML) membrane-encased NP (AMCNP) that carried the same surface antigen as source leukemia cells and used it as an anti-cancer vaccine (Fig. 19A). By modifying the cell membrane of C1498 mouse AML, the membrane-bound OVA (C1498-MovA) was identified as the model antigen, the NPs were coated with C1498-MovA membrane, CpG oligonucleotides were carried together as an immunostimulating adjuvant, and C1498-MOVa-AMCNPs with core-shell structure were formed. The NPs retained AML-specific antigens and effectively stimulated the cross-expression of DC-OVA-MHC class I after inoculation in vivo, inducing enhanced antigen-specific immune response and improving the immune response to AML [127]. Ochyl et al. prepared PEGylation NPs (PEG-NPs) from endogenous cell membranes collected from cancer cells. Combined with αPD-1 immunotherapy, the PEG-NP vaccine triggered a higher antigen-specific T cell response and played a role in mediating complete tumor regression in 63% of mouse models (Fig. 19B). In addition, combination immunotherapy with PEG-NP and αPD-1 protected all survivors from subsequent reattack by tumor cells [128].
6. *Bacterial outer membrane vesicles (OMVs):* OMVs refer to spherical natural vesicles usually generated from the outer membrane exhalation of Gram-negative bacteria. OMVs inherit various pathogenic molecular patterns from bacteria, like lipopolysaccharide, peptidoglycan, protein, and nucleic acid; therefore, they can effectively activate the immune system [226, 227]. Natural OMVs possess immunostimulating effects and tumor-resistant properties, hence are capable of stimulating the anti-tumor immune response mediated by IFN-γ [228]. Therefore, Li et al. focused on the fusion and expression of the extracellular region of PD-1 molecule on the OMV surface, which exerted an effective stimulating effect on the immune system and an inhibitory effect on immune cell depletion by tumor cells, thus strengthening the anti-tumor immune effect [129]. Zou et al. hybridized OMVs with tumor-derived cell membranes (mT) to form new functional vesicles (mTOMV). mTOMV caused innate immune cells to be further activated and strengthened the specific lysis ability of T cells in homogeneous tumors. In vivo, mTOMV could effectively accumulate in the inguinal lymph nodes and inhibit lung metastasis. This functional vesicle was biocompatible, had a simple preparation procedure, and could inhibit tumor growth and metastasis, making its application in clinical practice promising (Fig. 19C) [130].

### Preparation and mechanism of biomaterials

#### Preparation of biomaterials

##### Liposomes

Liposomes, originally called mesophones, are single- or multi-layer spherical vesicles that consist mainly of phospholipids from plants or animals. Upon the dispersion of phospholipids in water-based media, closed vesicles that have concentric lipid bilayer and hydrophilic core can be spontaneously formed. Liposome sizes range from 20 nm to several microns [229, 230]. Given the amphiphilic properties of the lipid components in liposomes, they can be used as candidate carriers for drug delivery. In a water-based environment, amphiphiles disperse, and when the concentration exceeds a certain limit, aggregation occurs by increasing the entropy of the system [231].

Liposomes have been long-investigated as underlying carriers for immunotherapy. Lai et al. synthesized specific TRP2180-188 peptide-loaded liposomes and adopted DC-targeted mannose and immune adjuvant CpG-ODN for the modification. These liposomes led to a substantial increase in tumor antigen-specific CD8^+^ cytotoxic T cells, thereby restricting tumor formation and proliferation [232]. Le et al. reported a remarkable mRNA delivery system consisting of cationic liposome (L), cationic polymer (P), and mRNA (R) that promoted DC targeting and produced a satisfactory antitumor response [233]. Chen et al. developed a lipid NP tumor vaccine that could strengthen the cross-presentation of antigens and promote STING activation; the vaccine produced a significant effect in the treatment of a mouse melanoma model [234]. Among all kinds of biological materials, liposomes have unique advantages of safety and effectiveness; therefore, they have been one of the most rapidly developed biological materials in recent years.

A variety of conventional methods are available for lipid regimes. Despite the ease of operation, conventional methods require massive amounts of organic solvents, and thus can cause adverse effects to human health and the environment. In addition, the residual organic solvents require complete elimination. The commonly used methods include the following: 1. Mechanical agitation: Powerful mechanical agitation (using an ultrasonic probe) is used to directly dissolve phospholipids in water [235]. This method is simple and feasible; however, the resulting small-sized liposomes present instability, which, together with drug leakage and preparation instability, limit the application of the produced liposomes in drug delivery. 2. Membrane hydration: Also known as the Bangham method [236], this technique uses evaporation to remove organic solvents to form lipid films, a time-consuming process. Afterwards, by means of agitation, the lipid film and water-based medium are hydrated to separate the swollen wafer from the container surface and form a closed spherical structure. This method is widely used and easy to operate; however, the dispersed phospholipids can produce multilayer liposomes with large shapes and sizes (1–5 μm in diameter) in the water-based buffer. Therefore, techniques that reduce the liposome size, such as ultrasound treatment to form small monolayer liposomes or LUV231 through a polycarbonate filter, are required. 3. Reverse phase evaporation method: Phospholipid and organic solvents are added to a round bottom flask. A rotary evaporator is adopted to remove solvents under reduced pressure and controlled temperature. The prepared liposomes are resolved in an organic phase after nitrogen purging, thereby increasing the drug encapsulation rate [237, 238]. 4. Solvent injection method (ethanol and ether): After the dissolution of the phospholipid in the organic solvent, the resulting solution is fully mixed with a water-based medium that contains the drug to be encapsulated in the liposome. The organic phase-aqueous phase interface presents the arrangement of lipids as monolayers, significantly assisting in the formation of a lipid bilayer [239] 5. Stain remover cleaning method: This uses the principle of stain remover-lipid micelle formation. When the stain remover is removed from the micelles, liposomes are formed. The removal rate and ratio of the remover to the lipid decides the liposome shape and size. However, the liposome concentration in the dispersion decreases, drug encapsulation rate decreases, and stain remover cannot be completely removed from the final preparation [237]. Large-scale preparation methods of liposomes include heating, freeze-drying, supercritical fluid method, supercritical fluid reverse evaporation method, micro-jet method, film contactor method, extrusion method, high-pressure homogenization method, and electric formation method [236].

##### Microspheres

Microspheres are spheres in which the particle sizes are in microns. Drug-carrying microspheres in the field of drug delivery are tiny spheres or spheroids that are formed by drug dissolution and dispersion in polymer materials, with particle size generally ranging from 1 to 250 μm. Microspheres load drugs by physically embedding or absorbing them on the surface or inside the polymer, and polymer stability guarantees the slow release effect of drugs [240, 241]. Microspheres have the advantages of long-term performance, safety, targeting, and high efficiency [240, 242]. Based on structure, microspheres can be categorized into pore microspheres, double-layer microspheres, and magnetic microspheres. Microspheres can be prepared by emulsification and volatilization, phase separation, spray-drying, and hot melt extrusion [243-246]. 1. Emulsified volatile method: This method is the most widely used. Fat-soluble drugs are prepared by oil-in-water emulsion and water-soluble drugs are prepared by the double-emulsion oil-in-water method. However, this method has many aspects that can affect the quality of microspheres and high research and development costs [247]. 2. Phase separation method: The agitated polymer-drug-solvent system is added with a third component (organic non-solvent), causing decreased solubility of the polymer, thereby creating the two phases at a certain point. Phase separation of the solvent and polymer is followed by the formation of a very ductile drug-loaded droplet. The system is then transferred to another organic non-solvent, and the microspheres are cured to obtain the final microsphere [246]. 3. Spray-drying method: The liquid raw and auxiliary materials are sprayed into the hot drying medium and converted into dry powder in the spray-dryer. The method can be applied to various drugs; however, the control of particle size of microspheres is poor [245]. 4. Hot melt extrusion method: The mixture of raw materials and auxiliary materials can be directly hot melted and extruded without dissolving the raw materials. The method portends no residual solvent problems, is safe and non-toxic, with an encapsulation rate close to 100% [245, 246]. Furthermore, new microspheres can be prepared through microfluidic technology and membrane emulsification technology. Microfluidic technology can control the microsphere particle size by changing the flow velocity; however, the production efficiency needs improvement [247]. Although membrane emulsification technology is simple to use, the prepared microspheres are too homogeneous in size [248]. Locally and globally, microsphere technology is developing rapidly and has great future potential.

##### Dendritic macromolecules

Dendritic macromolecules are macromolecules with a dendritic structure, which are repeatedly and linearly connected by oligomers through dendritic units. They usually contain a core, polymeric backbone chain as well as a side chain of dendritic units, and are dendritic monodisperse polymers. They grow repeatedly, which results in the formation of dendritic structures. With the increase of polymerization algebra, the degree of dendritic expansion continues to form a closed 3D spherical structure [249]. Representative dendritic macromolecules include polyamide-amine, poly-lysine, PEI, and polypropylene imine. For dendritic macromolecules, their physical and chemical properties are precise and controllable, internal cavity structure is extensive, and surface active functional groups are very intense. However, most of them are non-degradable [250]. Therefore, as a common drug delivery system, they face an inevitable risk of accumulation in the body, which may lead to cytotoxicity. Hence, the current development strategy of dendritic macromolecules is modification to reduce cytotoxicity or make them degradable [251]. The common synthesis routes for dendritic macromolecules are divergent and convergent growth methods; the convergent growth method has a good lowering side reaction probability and reagent amount but easily eliminates the functional activity of dendritic macromolecules [252]. Efficient synthesis routes include double exponential growth, double stage convergence, and supermonomer methods. Starpharma, an Australian company, has developed a dendrimer enhanced product technology platform for drug encapsulation and delivery by dendritic macromolecules, which can improve the efficacy of cancer treatment, lower drug toxicity and side effects, lengthen drug action time in vivo, and enhance targeted drug delivery. Dendritic macromolecules have the potential to become mainstream drug delivery systems, allowing the flourishing of related materials, preparation technologies, delivery methods, and delivery routes [253].

##### Microneedles

Microneedles are a new kind of physical penetration promotion technology, composed of multiple micron-level tiny tips linked to the base in arrays. The height and width of the needle body is 10–2000 and 10–50 microns, respectively. It allows the individual adjustment of the length, size, and shape based on therapeutic needs [254]. Microneedles can directly pass through the cuticle to generate micron-sized mechanical channels and place drugs in the epidermis or upper dermis to allow them to participate in microcirculation and bring about pharmacological reactions [255]. In 1998, Henry et al. first applied microneedles in transdermal drug delivery research. Hence, microneedle technology was introduced in the formal drug delivery field, triggering a development boom of microneedles and assisting in industrialization [255]. Based on their characteristics, there are solid, hollow, coated, soluble, and hydrogel microneedles [256]. Because of the difference in the properties of selected materials, different kinds of microneedles need to adopt different preparation technologies, such as chemical etching technology, electrical system processing technology, laser technology, and injection molding technology. Microneedles deliver drugs to the epidermis in four ways: first tie and then stick (solid microneedles), first tie and then release (soluble and frozen microneedles), coated delivery (coated microneedles), and injection delivery (hollow and gel microneedles). Chemical etching technology, micro electro mechanical system processing technology, and laser technology are generally used to prepare microneedles with strong mechanical force, and are mostly used to prepare solid, hollow, and coated microneedles. Injection molding technology is mainly used to prepare gel and soluble microneedles [254-257]. The application of microneedles in cancer immunotherapy by combining microneedle technology with drug delivery is still in the scientific research stage and has not yet been sufficiently developed [255, 257].

##### Hydrogels

Hydrogels are a kind of polymer with a 3D network structure, containing hydrophilic groups and water, with the water content usually ranging from 50 to 99.9%. Hydrogels have good biocompatibility, cross-linked network regulation, response to outside stimuli (pH, light, and temperature), molecular permeability, and other characteristics [258, 259]. They have gelatinous properties, thus serve as antigen storage caverns and carriers of cytokines, proteins, DNA, etc. Researchers reported injectable gels based on alginate microfin about a decade ago, which could be used for the co-delivery of mature redcs and chemokines CCL21 and CCL19 [259]. As observed, such hydrogel systems can recruit host DCs to the injection site, while simultaneously migrating to local lymph nodes, thereby ensuring a continuous process for the initiation of an immune response. The combination of tyramine HA hydrogel and sorafenib delivers IFN-α to the injection site and restrains tumor proliferation [260]. DNA-based supramolecular hydrogels release high concentrations of CpG, allowing the recruitment and activation of APCs, thereby contributing to tumor immunotherapy [261]. Currently, free radical copolymerization crosslinking is a representative method for designing and preparing medical polymer hydrogels [262].

##### Albumin NPs and albumin-binding NPs

Albumin NPs: Drugs are packaged in albumin NP delivery systems and can be attached to the albumin spatial structure via covalent binding or to the delivery system surface via physical adsorption. Furthermore, there are more advanced technologies for doping drugs into the NP matrix.

Albumin-binding NPs: These are the functional fragments of albumin-binding antibodies [263]. When combined with albumin, they extend the half-life and enhance the immune response to the polypeptide antigen. The preparation methods of albumin NPs are as follows: 1. Solvent removal method: The organic solvent is dropped into the albumin solution. As the proportion of organic solvent increases, the solubility of albumin weakens, resulting in denaturation and aggregation. Moreover, the addition of a crosslinking agent leads to condensation of the amino part of a lysine residue and guanidine part of arginine with the aldehyde group to solidify the albumin and form NPs. Finally, the organic solvent and residual crosslinking agent are removed, NPs are purified, and albumin NPs are collected [264]. 2. Emulsification method: Albumin aqueous solution and pharmaceutical aqueous solution are added to the oil phase or organic solvent containing an appropriate amount of emulsifier, and emulsified by stirring and ultrasonic or high pressure homogenization to form a water-in-oil emulsion. Subsequently, the emulsified drops are solidified by chemical crosslinking or heating deformation. Finally, the residual organic solvent is removed and albumin NPs are collected [265]. 3. Gel method: This includes hot gel method and pH gel method. Hot gel method is used to denature and aggregate albumin to form NPs under heating conditions. The pH gel method is used to precipitate albumin into NPs by adjusting the pH of the solution system [265]. 4. Self-assembly method: Paclitaxel for injection (albumin-binding) can be combined with PD-1 or PD-L1 antibodies for BC treatment. Although the current albumin NP delivery system can deliver drugs efficiently under the effects of retention and Gp60-mediated active targeting, further optimization of the delivery system is possible (Table 3) [266].

**Table 3:** Summary of preparation methods of various biomaterials

Biomaterials	Preparation methods
Liposome	Mechanical stirring, membrane hydration, reverse phase evaporation, and solvent injection
Microsphere	Emulsion volatilization, phase separation, spray-drying, and hot melt extrusion
Dendrimer	Divergent growth, convergent growth, double exponential growth, and double stage convergence method
Microneedle	Chemical etching, micro-electromechanical system processing, laser and mold injection
Albumin nanoparticles	Desolvent method, emulsification method, gel method, spray method

### Mechanism of biomaterials in immunotherapy

#### Enhanced antigen release and presentation

**Fig. 20:**
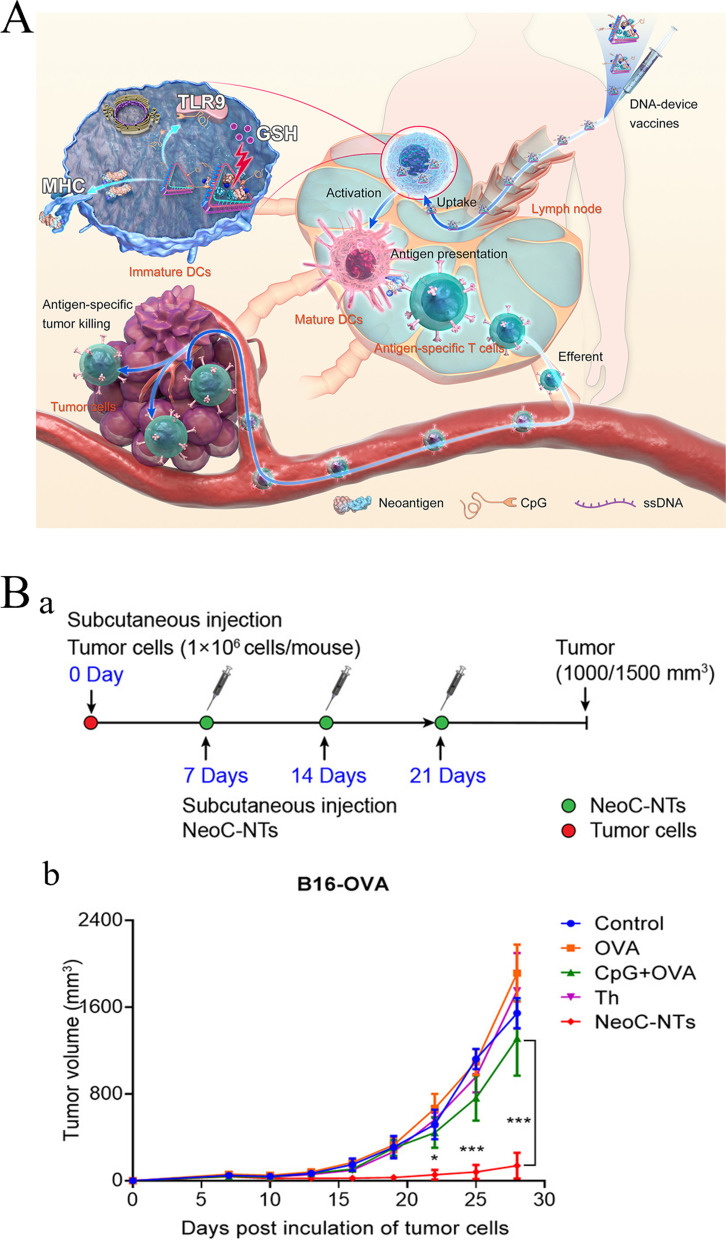
Schematic diagram of sulfonium-driven neoantigen-released DNA nanodevice. **A** Schematic Depictions of DNA-Peptide Conjugate Preparation and Tumor-Killing Response Induced by DNA Nanodevice Vaccines. **B** DNA nanodevice vaccines for personalized immunotherapy. (a) Administration plans for tumor-bearing C57BL/6 mice. (b) Average B16-OVA tumor growth curves. (Reproduced with permission from [271] Copyright © 2022, American Chemical Society)

Antigen release and presentation is a prerequisite for initiating cellular immunity. All processes, including the release of tumor antigen, capture, migration, and maturation of DCs, processing of antigen, and presentation of antigen to immature T cells through histocompatibility complex molecules, play crucial roles in initiating T cell-mediated immune responses [267]. Therefore, it is feasible to design and develop drug vectors for either step to enhance immune efficacy. However, many tumors have poor immunogenicity because of underexpression, loss, or downregulation of antigens [268]. Hence, most researchers focus on developing tumor nanovaccines capable of inducing the production of endogenous tumor antigens from the source or by directly delivering exogenous tumor antigens, supplementing the amount of antigens, and enhancing their “release and presentation” [269, 270]. Recently, to improve antigen-delivering efficiency and control release, Zhang et al. prepared a matte-driven DNA nanodevice that released neoantigens. The nanoplatform clearly demonstrated excellent stability, safety, and efficacy, and enhanced cytokine secretion, showing an effective inhibitory impact on melanoma growth and metastasis of lung melanoma (Fig. 20A, B) [271]. Li et al. also prepared a hybrid nanovaccine PTh/MnO2@M, which strengthened the T cell immune response by promoting antigen presentation, resulting in an effective and specific cancer therapy [272]. In recent years, biological materials for antigen release and presentation have emerged in large numbers and show much promise.

#### Promotion of T cell initiation and activation

T cells mainly drive acquired immune response and play an indispensable role in tumor immunotherapy. Their immune activation ensures normal downstream immunomodulatory response. Immature T cells take part in specific immune response at tumor sites after being activated by antigen recognition and co-stimulatory signals with APCs [273]. Presently, most immunotherapy strategies are often combined with nanocarriers through physical encapsulation, chemical grafting, and other methods for target delivery to the tumor site, inhibition of TRP catabolism, and catalysis of the growth of essential G1 phase T cells [274]. In addition, cytokines and chemokines (IL-2 and IFN-α/β) crucially affect the activation of T cells [275, 276]. A considerable number of nanobiomaterials, including ZnO and lipid NPs, have been proven to effectively promote cytokine secretion and T cell initiation and activation in vivo [3]. Zhang et al. developed a simple nanovaccine with C1 LNPs, which could effectively deliver mRNA in APCs. It could also activate TLR4 and induce robust T cell activation and inflammatory cytokine expression, showing obvious antitumor action in both tumor preventive and therapeutic vaccine settings [277].

#### Promotion of transport infiltration/recognition of CTLs

The last step of the tumor immune cycle is the transport infiltration/recognition killing process of CTLs. After activation, CTLs enter the tumor site through blood, and, by virtue of T cell antigen receptor interacting with histocompatibility complex molecules, complete specific recognition of tumor cells together with the release of granulozyme and perforin to eliminate tumor cells [273]. In immunosuppressed TME, several factors limit the smooth progression of this process. In view of these factors, functional immunomodulatory nanocarriers can be reasonably explored to synergistically enhance the therapeutic effect while delivering immune drugs in a targeted manner. Biomaterials that control tumor vascular normalization may be used to develop nanocarriers improve immune efficacy [278]. For example, some high-polymer structures containing nitric oxide (NO) donors may provide NO in TME, thereby regulating angiogenesis and maintaining vascular homeostasis. The release of NO gas in situ is accompanied by improved T cell-homing at the tumor site, which indirectly elevates the number of T cells in TME [279].

#### Regulation of TME

TME refers to the internal and external tumor environment, comprising many inflammatory cytokines/chemokines and immunosuppressive cells. Lymphocytes infiltrate the tumor for the mediation of the immunosuppressive TME, immune escape of tumor cells, and malignant tumor development, of which the effect largely depends on TAMs and Tregs. TAMs usually exhibit the M2 phenotype and exert antitumor activity [280, 281]. Based on this, some NPs have been developed for the alleviation of TME immunosuppression as well as enhancement of the anti-tumor immune response. Kim et al. constructed a bioactive glass NP BGN (F) with folic acid functionalization. Folic acid on the NP can effectively target the M2 phenotype TAM and release Si^4−^/Ca^2+^ to repolarize the M1 phenotype, which has a killing effect on tumors [282]. Zhang et al. utilized the inherent properties of black phosphorus for conducting tumor immunotherapy through a black phosphorus-based "minimalist" regimen. Black phosphorus-based PTT was capable of directly killing tumor cells, releasing tumor-related antigens, and stimulating immune response, thereby alleviating TMW immunosuppression [283]. Yang et al. adopted a hydrogel/nanomaterial composite system to construct a dual slow-release drug system, which regulated the TME by controlling the release of Apatinib, CD47 antibody (aCD47), and CpG, strengthened the synergistic impact on the primary tumor, and prevented tumor metastasis [284]. The development of scientific and effective treatment strategies for TME is conducive to relieving intratumor immunosuppression, limiting tumor immune escape, and enhancing the efficacy of tumor immunotherapy.

#### Simulating TME

In addition to immunotherapy for the TME, the effect of other immunotherapies also depends on the TME to a large extent, but the shortage of previous models simulating TME has greatly slowed the progress of cancer immunotherapy [285]. The emergence of 3D tumor models has effectively solved some of the problem. 3D tumor models mainly include bionic scaffold models, hydrogel models, organ-like models, organ chips, and scaffold-free models. Compared with the 2D model, the 3D model can more realistically simulate TME, which can be used to test immune-related therapies and shorten the drug development cycle [286]. Florczyk et al. prepared 3D porous chitosan alginate (CA) scaffold for culturing human prostate cancer cells to study the interaction between tumor cells and human peripheral blood lymphocytes in vitro. The experimental results showed that both matrix and CA scaffold supported the interaction between the lymphocytes and tumor cells, and CA scaffold could monitor and screen immunotherapy in situ more timely than matrix or other hydrogels [287]. In addition, Marrella et al. proposed an in vitro model of a 3D alginate-based hydrogel to evaluate the immunological characteristics of neuroblastoma and related personalized immunotherapy [288]. The 3D macro scaffold could observe the dynamic interaction process between biomaterials and tissues or organs to adjust the parameters any time [285, 289]. In recent years, 4D printing technology has been developed to include a time dimension on the basis of 3D structure. The shape or performance of 3D stents can change with time; therefore, 4D technology can be used to realize interventions in different time periods and further investigate the effect of tumor immunotherapy. 4D printing technology has great potential for the future [290].

## Conclusions

Tumorigenesis and the immune escape mechanism have been increasingly investigated and biotechnology has developed significantly, leading to improvements in tumor immunotherapy approaches. Tumor immunotherapy faces several challenges, such as poor specificity, low immunogenicity, inadequate delivery efficiency, and off-target side effects; however, the use of some biomaterials to improve anti-tumor immune effect of immunotherapy and control immunosuppression is promising. After closely combining with tumor immunotherapy, these biomaterials adopt the material carrier to avoid the degradation of therapeutic proteins and nucleic acids, increase the targeting of cytokines and genes, assist negative nucleic acids and cells to cross the barrier in vivo to reach the target, effectively reduce the systemic side effects caused by monoclonal antibodies, and achieve slow release in vivo.

To further promote the application of NPs and other biological materials in tumor immunotherapy, future studies should focus on the following four points: 1. Biological materials are foreign substances relative to the human organism. A resulting acute inflammation or chronic inflammation in the body may occur because of the biological material degradation process. Therefore, the safety and effectiveness of these biological materials need further evaluation. 2. The efficacy of biomaterial-based tumor immunotherapy designed for a single target is often insufficient; therefore, multiple approaches need to be combined to further improve the efficacy. Co-treatments that combine immunotherapy, chemotherapy, PTT, and radiation therapy should be strengthened for the integration of different tumor treatment methods. 3. There should be continued exploration of new tumor immune drugs, nanocarriers, and cell membranes, and the designing of the best load combinations and multi-drug co-transport systems according to their respective characteristics to achieve higher encapsulation rates and controlled release abilities. 4. There are still many tumor immunoregulation-based pathways that have not yet been developed as corresponding immunotherapy strategies. Further research on tumor immunotherapy and nanodelivery mechanisms is recommended to improve these innovative strategies in the future to provide diversified tumor therapy programs.

In conclusion, research on biomaterial-based tumor immunotherapy is booming; however, several challenges remain to be overcome to transition from experimental research to clinical application. Biomaterials have been optimized continuously and nanotechnology has achieved continuous progression, ensuring the development of more efficient biomaterials, thereby providing a platform and opportunity for breakthroughs in tumor immunotherapy.

## Data Availability

Not applicable.

## Abbreviations

ACT: Adoptive cell therapy
AML: Acute myelocytic leukemia
AMLCNP: AML membrane-encased nanoparticle
APC: Antigen-presenting cell
BC: Breast cancer
BPQD: Black phosphorus quantum dots
bsAb: Bispecific antibody
CA: Chitosan alginate
CAR: Chimeric antigen receptor
CDNs: Circular dinucleotides
CIK: Cytokine-induced killing
CNT: Carbon nanotube
CpG: Guanine cytosine phosphate
CTLA-4: Cytotoxic T lymphocyte-associated antigen-4
DC: Dendritic cell
DNCaNPs: PLGA-CaCO_3_ NPs loaded with DOX/aNLG919
DOX: Doxorubicin
FDA: Food and Drug Administration
HA: Hyaluronic acid
ICD: Immunogenic cell death
ICI: Immune checkpoint inhibitor
IFN: Interferon
i-GMD: Injectable CAR-T geline-methacrylate hydrogel delivery
IL: Interleukin
LNDs: Lipid nanodisks
MDSC: Myeloid-derived suppressor cell
MRI: Magnetic resonance imaging
MWCNTs: Multi-walled carbon nanotubes
NK: Natural killer
NP: Nanoparticle
NRT: Neoantigen-reactive T cell
NSCLC: Non-small cell lung cancer
OMV: Outer membrane vesicle
OV: Oncolytic virus
OVA: Ovalbumin
PDNP: PH-activated supramolecular nanoprodrug
PLLA: Poly-lactic acid
PD-1: Programmed death-1
PDNPs: PH-activated supramolecular nanoprodrugs
PDT: Photodynamic therapy
PEG: Polyethylene glycol
PEG-SAB-Lip: Salvianolic acid B-loaded PEGylated liposomes
PEI: Polyethylenimine
PLA: Polylactic acid
PLG: Polylactic acid-glycolic acid
PTT: Photothermal therapy
R848: Resiquimod
RBC: Red blood cell
ROS: Reactive oxygen species
SCM: Stem cell membrane
SF: Silk fibroin
STB: Shear-thinned biomaterial
STING: Stimulator of interferon genes
TAM: Tumor-associated macrophage
TAMM: Tumor-associated macrophage cell membrane
TCR: T cell receptor
Th: T helper
TIL: Tumor-infiltrating lymphocyte
TLR: Toll-like receptor
TME: Tumor microenvironment
TNBC: Triple negative breast cancer
Treg: Regulatory T cell
TRP2: Tyrosinase-related protein 2
ZNO NPs: Zinc oxide NPs
